# Energy Deficiency in Soldiers: The Risk of the Athlete Triad and Relative Energy Deficiency in Sport Syndromes in the Military

**DOI:** 10.3389/fnut.2020.00142

**Published:** 2020-08-25

**Authors:** Thomas J. O'Leary, Sophie L. Wardle, Julie P. Greeves

**Affiliations:** ^1^Army Health and Performance Research, Army Headquarters, Andover, United Kingdom; ^2^Division of Surgery and Interventional Science, UCL, London, United Kingdom; ^3^Norwich Medical School, University of East Anglia, Norwich, United Kingdom

**Keywords:** energy availability, energy deficit, endocrinology, physical performance, reproductive function, bone

## Abstract

Military personnel experience energy deficit (total energy expenditure higher than energy intake), particularly during combat training and field exercises where exercising energy expenditures are high and energy intake is reduced. Low energy availability (energy intake minus exercising energy expenditure expressed relative to fat free mass) impairs endocrine function and bone health, as recognized in female athletes as the Female Athlete Triad syndrome. More recently, the Relative Energy Deficiency in Sport (RED-S) syndrome encompasses broader health outcomes, physical and cognitive performance, non-athletes, and men. This review summarizes the evidence for the effect of low energy availability and energy deficiency in military training and operations on health and performance outcomes. Energy availability is difficult to measure in free-living individuals but doubly labeled water studies demonstrate high total energy expenditures during military training; studies that have concurrently measured energy intake, or measured body composition changes with DXA, suggest severe and/or prolonged energy deficits. Military training in energy deficit disturbs endocrine and metabolic function, menstrual function, bone health, immune function, gastrointestinal health, iron status, mood, and physical and cognitive performance. There are more data for men than women, and little evidence on the chronic effects of repeated exposures to energy deficit. Military training impairs indices of health and performance, indicative of the Triad and RED-S, but the multi-stressor environment makes it difficult to isolate the independent effects of energy deficiency. Studies supplementing with energy to attenuate the energy deficit suggest an independent effect of energy deficiency in the disturbances to metabolic, endocrine and immune function, and physical performance, but randomized controlled trials are lacking.

## Introduction

Military personnel experience episodes of energy deficit (total energy expenditure higher than energy intake) throughout their career. High exercising energy expenditures and restricted food intake, either due to logistical constraints, suppressed appetite, or as part of a training objective, are contributing factors (1–8). Prolonged periods of energy deficit can negatively impact health and performance (9–11) and potentially impair military effectiveness. The Female Athlete Triad (Triad) (11) and Relative Energy Deficiency in Sport (RED-S) (9, 10) syndromes describe the effects of chronic low energy availability (energy intake minus exercise energy expenditure expressed relative to fat free mass) (12) on health and performance outcomes. The Triad reflects the effect of low energy availability on menstrual disturbances and low bone mineral density (BMD) (11); RED-S describes the effects of low energy availability on a range of wider health and performance outcomes in “at risk” sporting and some non-sporting (e.g., dancers) civilian populations (9, 10). The relevance of the Triad and RED-S in a military context has not been comprehensively reviewed.

The physical and psychological challenges faced by military personnel are inherently different than those faced by athletes. The implications of impaired performance are also different between military and sporting populations; military tasks require a unique combination of physical and cognitive effort in unpredictable and stressful environments, and the consequences of underperformance can be catastrophic. The predisposing factors leading to, and health and performance implications of, low energy availability in sport, may, therefore, not be applicable to the military. Training for sport is focussed on optimizing performance and often a desire for “leanness,” whereas military training prepares individuals for the hostile physical and psychological conditions of combat (e.g., concomitant periods of prolonged exercise, food restriction, sleep deprivation, extreme environments, and psychological stress). Evidence for the effects of low energy availability in the Triad (11) and RED-S (9, 10) mostly originates from short-term laboratory studies and cross-sectional studies comparing amenorrhoeic and eumenorrhoeic female athletes. Longitudinal prospective assessment of low energy availability is possible in military populations in the field, where energy restriction is often induced purposively as a training objective or due to logistical constraints. Whilst military field studies are not designed for this purpose, and involve exposure to several stressors, data from these studies contribute to an understanding of the implications of low energy availability on health and performance. Most evidence for the effects of low energy availability presented within the Triad (11) and RED-S (9, 10) are for clinical outcomes, and a better understanding of performance outcomes has important implications for the military. The aim of this article is to review the evidence for the effect of energy deficiency on health and performance outcomes implicated in the Triad and RED-S in military populations.

## Energy Availability and Energy Balance

Energy availability is the dietary energy available for metabolic function after exercise, defined as energy intake minus exercise energy expenditure expressed relative to fat free mass (FFM) (Equation 1) (12). Dietary energy is essential for physiological processes, including locomotion, thermoregulation, reproduction, and growth (13). High exercise energy expenditures (locomotion) during athletic or military training use energy that is no longer available for other processes (12). Low energy availability partitions metabolic fuels toward processes essential for survival (i.e., circulation and neural activity) over non-essential processes (i.e., reproduction and growth) (13). The mechanism is through altered endocrine signaling from the central nervous system (i.e., decreased release of gonadotropin releasing hormone to suppress reproductive function) in response to acute changes in cellular fuel oxidation and peripheral hormones (13). This altered endocrine signaling can be harmful to health and performance.

Energy availability exists on a spectrum from optimal to low, with or without disordered eating, as highlighted by the Triad (11). Energy availability for normal metabolic function is 45 kcal·kg FFM^−1^ · d^−1^ (12, 14–18). Energy availability below 30 kcal·kg FFM^−1^ · d^−1^, equivalent to resting metabolic rate (12), decreases luteinising hormone (LH) pulse frequency, oestradiol, 3,3,5-triiodothyronine (T3), insulin-like growth factor-1 (IGF-1), and leptin, and increases cortisol, growth hormone (GH), and bone resorption, in women (14, 15, 17). Low energy availability has, therefore, been considered as < 30 kcal·kg FFM^−1^ · d^−1^ in the Triad (11, 19) and RED-S (9, 10). Although defining a threshold for low energy availability is practically useful, the effect of differing severities of low energy availability is likely dependent on the physiological system, and an individual's body size, age, and sex (10); further validation of this threshold against other clinical outcomes is required.

*Energy Availability [kcal*·*kg FFM*^−1^ · *d*^−1^*] = (Energy Intake [kcal*·*d*^−1^*]—Exercise Energy Expenditure [kcal*·*d*^−1^*])/Fat Free Mass [kg]*.

Equation 1. Calculation of energy availability (12).

Energy balance is defined as energy intake minus total energy expenditure (i.e., exercise and all other physiological processes) and is distinctly different from energy availability; energy availability is the input to physiological processes, whereas energy balance is the net change in total body energy stores resulting from the output from physiological processes (12). The acute metabolic adaptations that occur with low energy availability reduce resting metabolic rate and total energy expenditure, and can result in an individual having low energy availability despite being in energy balance and body mass stable (12, 19). Most military studies have measured energy balance or body mass to estimate energy status (reviewed in Demands of Military Training and Employment); for the purpose of this review, energy deficit is used to reflect a negative energy balance rather than low energy availability. Findings from these military studies must, therefore, be interpreted in context of these definitions and physiological implications.

### Female Athlete Triad and Relative Energy Deficiency in Sport

The observation that disordered eating in female athletes, particularly in sports emphasizing “leanness,” leads to functional hypothalamic amenorrhoea and osteoporosis, was first summarized by the American College of Sports Medicine with the Triad framework in the 1990s (20). The Triad was updated in 2007 to reflect the inter-relationship of energy availability (with or without disordered eating), menstrual disturbance (with or without functional hypothalamic amenorrhoea) and low BMD (with or without osteoporosis) on a spectrum (11).

Subsequent to the Triad, the International Olympic Committee defined RED-S in 2014 (9), with further updates in 2015 and 2018 (10, 21). The RED-S syndrome expands the Triad to include men, populations other than athletes (e.g., dancers), and other clinical and performance outcomes affected by low energy availability (9, 10), although the evidence surrounding some of the outcomes in RED-S and their clinical relevance has been questioned (22). In brief, the RED-S syndrome highlights that low energy availability can have effects on endocrine and metabolic function, menstrual function, bone health, immune function, hematological function, gastrointestinal health, psychological well-being, and physical and cognitive performance (9, 10):

*The syndrome of RED-S refers to impaired physiological function including, but not limited to, metabolic rate, menstrual function, bone health, immunity, protein synthesis, cardiovascular health caused by relative energy deficiency (**9**)*.

Disordered eating and/or the desire to be lean for aesthetic or performance reasons are considered pre-dispositions for the Triad (11) and RED-S (9). Unlike some sports, a specific body composition is not a prerequisite to successful military performance (23), however, leanness may be desired by some military personnel, militaries have body mass employment standards, and there is evidence of disordered eating in military women (24, 25). Environments with unavoidable extreme exercise and insufficient energy intake also likely contribute to the development of RED-S (9), and there is evidence of the Triad in non-athletes as well as athletes (26) and in men as well as women (27). It, therefore, seems prudent to question whether components of the Triad and RED-S are evident in military personnel, considering: (i) periods of sustained high exercising energy expenditures with restricted food intake (either as a training objective, due to limited food availability in hostile environments, or suppressed appetite) are imposed on military personnel, particularly in the more arduous combat roles; (ii) all military roles, including these arduous combat roles, are now open to both men and women in many militaries, and; (iii) military occupational tasks require the performance components proposed within RED-S.

## Demands of Military Training and Employment

### Physical Demands

Military tasks are diverse (e.g., combat field exercises, casualty extraction, weapon handling, repetitive lifting, prolonged load carriage) and impose a wide range of physiological stresses. Aerobic capacity and muscle strength, power, and endurance are all important for performing military tasks (28). Basic military training prepares new recruits for these tasks, and, therefore, consists of a range of activities including physical training (aerobic training, strength and conditioning, circuit training, obstacle courses, agility training, and swimming), field exercises, adventure training, and training on a variety of military specific skills including load carriage, marching, military drill, and weapon and equipment handling (29). Basic military training is physically demanding, and total energy expenditures can exceed ~3,400 and ~4,400 kcal·d^−1^ for female and male recruits (29–32); however, food is usually eaten *ad libitum*. Beyond basic military training, trained soldiers undertake specialist military training courses and field exercises in preparation for the hostile nature of combat; selection and field exercises, therefore, impose the added stressors of energy restriction, sleep deprivation, and psychological stress (2, 33). For the purpose of this review, basic military training refers to training courses completed by new recruits upon entry to the military, and specialist military training refers to advanced combat or promotional courses, or field exercises, completed by trained soldiers throughout their careers.

### Energy Availability and Energy Balance

The direct measurement of energy availability in free-living individuals is challenging, with no consensus on measurement protocols (e.g., defining what qualifies as exercise energy expenditure) and inherent inaccuracies with the required techniques (e.g., self-reported energy intake) (10, 34). The uniform nature of military training (e.g., same equipment, physical activity, and eating and sleeping patterns) may help standardize measurement of energy availability, but exercise energy expenditure is difficult to define in military training. There are various ambiguous definitions of exercise energy expenditure (i.e., purposeful training, other leisure activities, or activity above a certain intensity) (34), and unlike athletic training, military training does not comprise discrete bouts of physical activity.

Most military studies have measured energy balance rather than energy availability, likely due to the relative simplicity of measurement. Numerous studies have measured total energy expenditure and/or energy balance in military training and on operations with either doubly labeled water or dual energy X-ray absorptiometry (DXA). A comprehensive overview of these studies is presented in Table 1. These studies demonstrate that total energy expenditures, measured using the doubly labeled water method, are high, and largely differ between individuals as a function of body mass (29, 31, 49). Studies that have measured both total energy expenditure and energy intake have observed energy deficits (i.e., negative energy balance), but the measurement of energy intake is inherently inaccurate. Some studies have calculated energy deficits using DXA, which measures the change in fat mass and FFM based on the known energy densities of the two tissues (2, 4, 33, 53, 61). Food, either served in canteens or in ration packs, is often provided in standard portions or must be consumed within restricted times, and, therefore, some heavier individuals may not meet their energy requirements (1). In addition to high total energy expenditures, restricted energy intake, as the result of logistical barriers to eating or as a training objective (6), a hesitancy to carry extra weight (71), sub-optimal dietary practices (72, 73), or suppressed appetite or menu fatigue (6), may all contribute to energy deficits. The remainder of this review discusses the evidence for the health and performance implications of this energy deficiency in military personnel.

**Table 1:** Field studies measuring total energy expenditure and/or energy balance in military training and operations with doubly labeled water or dual energy X-ray absorptiometry.

**Military activity**	**Participants**	**Measurements**	**Total energy expenditure (kcal·d^**−1**^)**	**Energy intake (kcal·d^**−1**^)**	**Energy balance (kcal·d^**−1**^)**
Australian Air Defense Guards training (12 days) (35)	10 men provided full rations; 10 men provided half rations; 11 men provided fresh food	TEE: doubly labeled water over 7 days (*n =* 8); EI: weighed-food, estimations, and ration pack discards; EB: not assessed	3,650 ± 1,060	Full ration: 2,197 ± 549; half ration: 1,576 ± 191; fresh feeding: 2,866 ± 310	Not assessed
Australian Army jungle warfare training (12 days) (36)	34 men	TEE: doubly labeled water over 7 days (*n =* 4); EI: weighed-food, estimations, and ration pack discards; EB: not assessed	4,750 ± 531	4,040 (mess: 5,135; field: 2,582)	Not assessed
British Army basic training (12 weeks) (31)	7 women; 7 men	TEE: doubly labeled water over 10 days across week 1 to 2 and week 9 to 10; EI: not assessed; EB: not assessed	Women: 2,964 ± 263 and 2,988 ± 143; men: 3,633 ± 359 and 3,537 ± 335	Not assessed	Not assessed
British Army basic training (14 weeks) (32)	10 women; 9 men	TEE: doubly labeled water over 10 days across week 1 to 2 and week 13 to 14; EI: not assessed; EB: not assessed	Women: 2,986 ± 382 and 3,227 ± 454; men: 4,159 ± 621 and 4,350 ± 478	Not assessed	Not assessed
British Army basic training (14 weeks) (29)	17 women; 16 men	TEE: doubly labeled water over 10 days across week 1 to 2 and week 12 to 13; EI: not assessed; EB: not assessed	Women: 2,847 ± 323 and 3,390 ± 344; men: 4,020 ± 620 and 4,253 ± 556	Not assessed	Not assessed
British Army Infantry basic training (14 weeks) (37)	14 men	TEE: doubly labeled water over 10 days across week 1 and 2; EI: not assessed; EB: not assessed	4,419 ± 430	Not assessed	Not assessed
British Army Officer basic training (44 weeks) (38)	10 women; 10 men	TEE: doubly labeled water over 10 days; EI: not assessed; EB: not assessed	4,112 ± 652	Not assessed	Not assessed
British Army Parachute Regiment basic training (24 weeks) (30)	6 men	TEE: doubly labeled water over 10 days across week 1 to 2 and week 19 to 20; EI: not assessed; EB: not assessed	4,735 ± 700 and 4,696 ± 545	Not assessed	Not assessed
British Army Section Commanders battle course (8 weeks) (1)	27 men	TEE: doubly labeled water over 10 days across week 2 to 3 and week 6 to 7; EI: estimated from TEE and EB; EB: estimated from changes in FFM and FM measured with doubly labeled water	4,693 ± 424 and 5,094 ± 471	4,235	−644
British Army Section Commanders battle course (8 weeks) (4)	15 male controls; 15 men provided extra 1,218 kcal·d^−1^	TEE: not assessed; EI: not assessed; EB: estimated from changes in FFM and FM measured by DXA	Not assessed	Not assessed	Normal training: −526 ± 263; supplemented training: −167 ± 263
British Royal Marines on deployment to Afghanistan (6 months) (39)	18 men	TEE: doubly labeled water over 7 days mid-deployment; EI: 7-day food record mid-deployment; EB: not assessed	3,626 ± 450	Patrolling days: 2,194 ± 630; non-patrolling days: 2,095 ± 613	Not assessed
Canadian Infantry arctic field exercise (10 days) (40)	10 men	TEE: doubly labeled water over 7 days; EI: food records; EB: EI/TEE × 100	4,317 ± 927	2,633 ± 499	EI was 61% of TEE
Finish Defense Force basic training (8 weeks) (41)	24 men	TEE: doubly labeled water over 15 days during last 2 weeks of training; EI: food records over 7 days; EB: (EI—TEE)/TEE × 100	3,697 ± 394	2,752 ± 771	−26 ± 18%
Finish Defense Force basic training (8 weeks) (42)	24 men	TEE: doubly labeled water over 8 days during field exercise; EI: not assessed; EB: not assessed	3,965 ± 502	Not assessed	Not assessed
Gulf Cooperation Council country Army, Air Force and Navy Officer training (2 to 3 years) (43)	119 men	TEE: doubly labeled water over 7 days; EI: not assessed; EB: not assessed	3,057 ± 429 to 3,301 ± 504	Not assessed	Not assessed
Israeli Defense Force winter and summer Infantry training (44)	18 men in winter; 12 men in summer	TEE: doubly labeled water over 12 days (winter, *n =* 14; summer, *n =* 10); EI: food records; EB: EI—TEE	Winter: 4,281 ± 170^c^; summer: 3,937 ± 159^c^	Winter: 2,792 ± 108^c^; summer: 2,857 ± 179^c^	Winter: −1,422 ± 163^c^; summer: −924 ± 232^c^
Royal Netherlands Army submarine deploymemt (3 months) (45)	10 men	TEE: doubly labeled water over 2 weeks (weeks 4 to 5); EI: estimated from TEE and EB; EB: estimated from changes in FFM and FM measured with doubly labeled water	2,937 ± 498	3,158 ± 786	221 ± 506
Norwegian Army winter training (4 days training, 3 days ski march) (46)	21 men	TEE: doubly labeled water; EI: discards from ration packs; EB: EI—TEE	6,140 ± 394 (training: 5,480 ± 389; ski march: 6,851 ± 562)	Training: 3,098 ± 236; ski march: 3,461 ± 586	−2,899 ± 498 (training: −2,382 ± 499; ski march: −3,390 ± 669)
Norwegian Army winter training (4 days) (47)	2 women and 71 men (18 controls; 27 provided additional carbohydrate; 28 provided additional protein [both ~1,000 kcal·d^−1^])	TEE: doubly labeled water (controls, *n =* 14; carbohydrate, *n =* 14; protein, n =14); EI: food records; EB: EI—TEE	Control: 6,096 ± 412; carbohydrate: 6,181 ± 505; protein: 6,167 ± 592	Control: 2,506 ± 410; carbohydrate: 3,131 ± 633; protein: 2,825 ± 599	Control: −3,595 ± 606; carbohydrate: −3,050 ± 888; protein: −3,402 ± 687
Norwegian Defense Cyber Academy field exercise (10 days) (48)	4 women and 15 men provided low protein (1 g·kg·d^−1^), and 3 women and 16 men provided high protein (2 g·kg·d^−1^) diet	TEE: not assessed; EI: food provided; EB: estimated from changes in FFM and FM measured by DXA	Not assessed	Low protein: 1,183 ± 168; high protein: 1,174 ± 170	Low protein: −4,373 ± 1,250; high protein: −4,271 ± 1,075
Norwegian Ranger training (7 day field exercise) (49)	6 women; 10 men	TEE: doubly labeled water over 7 days; EI: estimated from food provided; EB: not assessed	Women: 5,234 ± 478; men: 6,358 ± 478	Women: 48 to 454; men: 48 to 526	Not assessed
US Army basic combat training (10 weeks) (50)	14 women; 30 men	TEE: doubly labeled water over 5 days during week 5 and 10; EI: not assessed; EB: not assessed	Women: 3,412 ± 350 and 3,325 ± 493; men: 4,279 ± 445 and 4,096 ± 500	Not assessed	Not assessed
US Army construction and humanitarian tasks at altitude (15 days) (51)	35 male controls; 32 men provided additional carbohydrate	TEE: doubly labeled water (*n =* 11); EI: visual estimations and food records; EB: not assessed	3,549 ± 608	Control: 2,140 ± 94; carbohydrate: 2,265 ± 119	Not assessed
US Army Ranger selection and assessment programme (6 weeks) (52)	131 men	TEE: doubly labeled water over 5 days during week 1 (*n =* 16); EI: estimated as food provided from menus (canteen) and ration packs (field); EB: not assessed	4,264 ± 342	2,919 ± 331	Not assessed
US Army Ranger training (8 weeks) (33, 53)	50 to 55 men	TEE: estimated from EB and EI; EI: estimated from rations provided; EB: estimated from changes in FFM and FM measured by DXA	2,796 to 3,220	3,991 to 4,200	−1,195 ± 390 to −900 ± 390
US Army Ranger training (8 weeks) (2)	49 male controls; 48 men provided extra 400 kcal·d^−1^	TEE: not reported; EI: estimated as food provided from menus (canteen) and ration packs (field); EB: estimated from changes in FFM and FM measured by DXA	Figure not reported	Figure not reported	Controls: −1,195 ± 478; supplemented: −980 ± 382
US Marine cold weather field exercise (11 days) (54)	23 men	TEE: doubly labeled water; EI: food records; EB: estimated from changes in FFM and FM measured with doubly labeled water	4,919 ± 190^c^	3,132 ± 165^c^	−1,872 ± 293^c^
US Marine desert field exercise (11 days) (55)	11 men provided carbohydrate drink; 8 men provided placebo drink	TEE: doubly labeled water; EI: food records and visual estimation; EB: not assessed	Carbohydrate: 4,397 ± 1,051; placebo: 3,950 ± 645	Carbohydrate: 3,415 ± 143; placebo: 3,057 ± 191	Not assessed
US Marine Infantry Officer training (7 day field course) (56)	8 men	TEE: doubly labeled water over 50 h; EI: empty food wrappers from rations; EB: not assessed	3,647 ± 394	1,366 ± 272	Not assessed
US Marine Infantry Officer training (8 day field course) (57, 58)	29 to 34 men	TEE: doubly labeled water (*n =* 12); EI: empty food wrappers from rations; EB: not assessed	3,834 ± 200 to 3,862 ± 200	1,540 ± 300 to 1,540 ± 235	Not assessed
US Marine Infantry Officer training (8 day field course) (59)	18 men provided low protein (0.5 g·kg·d^−1^) and 17 men provided moderate protein (0.9 g·kg·d^−1^) rations	TEE: doubly labeled water (low protein, *n =* 6; moderate protein, *n =* 12); EI: empty food wrappers from rations; EB: EI—TEE	Low protein: 3,941 ± 478; moderate protein: 3,798 ± 502	Low protein: 1,552 ± 143; moderate protein: 1,529 ± 167	Both groups: −2,317
US Marines Special Operation Command individual training (four phases [each 7 to 23 days] over 9 months) (60)	9 to 13 men	TEE: doubly labeled water; EI: 24 h dietary recalls and food records; EB: EI—TEE	3,754 ± 314 to 6,376 ± 712	346 ± 0 to 2,819 ± 488	−1,027 ± 740 to −3,966 ± 776
US Marine survival, evasion, resistance, escape training (18 days) (61)	63 men	TEE: not assessed; EI: not assessed; EB: estimated from changes in FFM and FM measured by DXA	Not assessed	Not assessed	−4,203 ± 1,686
US Marine winter field exercise (54 h) (62)	20 women; 30 men	TEE: doubly labeled water; EI: empty food wrappers from rations; EB: EI—TEE	Women: 4,729 ± 143; men: 6,138 ± 191	Women: 1,146 ± 430; men: 1,433 ± 478	Women: −8,121 ± 1,433; men: −10,318 ± 2,484 over 54 h
US Natick Soldier Research, Development and Engineering Center personnel during normal duties (7 days) (63)	2 women; 24 men	TEE: doubly labeled water; EI: written or personal digital assistant food records; EB: (EI—TEE)/TEE × 100	Written record group: 3,141 ± 647; personal digital assistant group: 3,158 ± 614	Written record group: 3,266 ± 635; personal digital assistant group: 2,865 ± 716	Written record group: 3%; personal digital assistant group: −8%
US Navy Sailors on amphibious assault ship (8 days) (64)	10 women; 7 men	TEE: doubly labeled water; EI: not assessed; EB: not assessed	2,998 ± 788	Not assessed	Not assessed
US Special Forces field training (28 days) (65)	8 men provided full rations (4,020 kcal·d^−1^); 8 men provided lightweight rations (1,980 kcal·d^−1^)	TEE: doubly labeled water over 28 days; EI: daily food records; EB: not assessed	Full ration: 3,480 ± 220; lightweight ration: 3,320 ± 280	Full ration: first 14 days 2,840 ± 280, second 14 days 3,080 ± 630; lightweight ration: first 14 days 1,900 ± 130, second 14 days 1,960 ± 120	Not assessed
US Special Forces hot and cold climate training (4 to 5 days) (66)	21 men in hot environment; 8 men in cold environment	TEE: doubly labeled water; EI: food recall; EB: EI—TEE	Hot: 4,664 ± 1,339; cold: 4,549 ± 1,221	Hot: 2,200 ± 711; cold: 3,001 ± 900	Hot: −2,464 ± 1,696; cold: −1,548 ± 1,607
US Special Forces pre-deployment and combat diver training (7 days) (67)	29 men	TEE: doubly labeled water; EI: not assessed; EB: not assessed	Pre-deployment training: 3,901 ± 521; combat diver training: 4,564 ± 351	Not assessed	Not assessed
US Special Forces training (6 day mountainous field exercise) (68)	6 men	TEE: doubly labeled water; EI: daily food records; EB: estimated from changes in FFM and FM, predicted from underwater weighing and total body water	4,554 ± 566	2,357 ± 860	−2,280 ± 368
US Special Forces training (four phases over 64 days) (3)	36 men	TEE: doubly labeled water over 10 days; EI: visual estimation (canteen) or discards from ration packs (field); EB: EI—TEE	3,633 ± 980 to 5,210 ± 717	Minimum of 2,510 ± 884; maximum not reported^a^	Up to −2,700 ± 540
US Special Forces training (routine garrison training) (69)	32 men in special forces; 13 male support soldiers	TEE: doubly labeled water over 9 days (special forces, *n =* 9; support soldiers, *n =* 9); EI: visual estimation and food records; EB: not assessed	Special forces: 4,099 ± 740; support soldiers: 3,136 ± 652	Special forces and support combined: 3,204 (2,838, 3,676)^b^	Not assessed
Zimbabwean Commando heat-stress field exercise (70)	8 men	TEE: doubly labeled water over 10 days; EI: food records; EB: not assessed	5,493 ± 358^c^	4,060 ± 358^c^	Not assessed

*Data are mean ± SD unless otherwise stated*.

a *Data estimated from figures*;

b *median and interquartile range*;

c *mean ± standard error*.

*DXA, dual energy X-ray absorptiometry; EB, energy balance; EI, energy intake; FFM, fat free mass; FM, fat mass; Mess, military canteen; TEE, total energy expenditure*.

## Energy Deficiency and Health

### Endocrine and Metabolic Function

Low energy availability decreases reproductive (gonadotropins and sex steroids), thyroid (T3), and metabolic (IGF-1, leptin) hormone concentrations, and increases cortisol and GH (14–17). Widespread disturbances in endocrine and metabolic function are evident in energy deficient soldiers during specialist military training. In Norwegian military cadets, 5 days of combat training increased circulating cortisol and GH, and decreased thyroid (T3 and thyroxine [T4]) and reproductive (follicle-stimulating hormone [FSH], testosterone, and oestradiol) hormone concentrations (74–79). The energy deficit was severe—estimated energy intake ≤ 1,600 kcal·d^−1^ and energy expenditure ≥ 8,500 kcal·d^−1^–and total sleep was ~2 h across the 5 days. Subsequent studies of male US Army Rangers support these findings, and demonstrate that 8 weeks of combat training in energy deficit (~1,000 kcal·d^−1^) increased cortisol, and decreased T3, IGF-1, and reproductive hormones (decreased LH and testosterone, increased sex hormone binding globulin [SHBG]) (2, 80–82). Disturbances to endocrine and metabolic function were also evident following a wide range of other specialist military training courses (4, 48, 57, 59, 83–85). Limited studies on operational deployment show decreased (with body mass losses) (86) or unchanged (with stable body mass) (87) reproductive hormone concentrations in men following deployment. The long-term effects of acute changes in endocrine function are unclear, but a cross-sectional analysis of experienced naval operators completing normal duties reported low testosterone suggesting chronic implications for gonadal function (88). Further work on the implications of these changes in sex steroids on reproductive function in men is required.

There are fewer studies on military women, likely due, in part, to their historic exclusion from combat roles and there is a greater proportion of men in the military. During a 5.5 day Norwegian Special Forces field exercise, women had a greater increase in cortisol and greater reduction in IGF-1 than men (89). The men and women did not, however, complete the same activities and further data comparing the response of men and women to the same military training are warranted. There is emerging evidence that female military personnel can experience low energy availability without effects on hypothalamic-pituitary-gonadal and adrenal axis function, assessed using clinically relevant dynamic testing (90). Six women who lost 13% body mass during a 61 day Antarctic crossing had largely unchanged responsiveness of the gonadal and adrenal axes, and metabolic status (90, 91). Results from this study provide important insight into extreme energy deficit in women but most outcomes were underpowered. For example, Korean female military cadets lost ~5% of body mass after 4 and 8 weeks of training and had decreased oestradiol (92). Assessment of hypothalamic-pituitary-adrenal axis responsiveness after adrenocorticotrophic hormone administration showed no mal-adaptation in women after 28 weeks of the 44 week British Army Officer training course, but increases in hair and saliva cortisol were observed in the initial weeks of training (93). Markers of psychological stress were also increased and so it is likely that increased cortisol was due, in part, to psychological stress. Whilst these observational studies in men and women demonstrate disturbed endocrine function, high levels of physical activity, sleep deprivation, and psychological stress could contribute to these responses and the role of energy deficit cannot be confirmed.

Several studies have examined differing severities of energy deficit and these studies provide support for a relationship between energy status and endocrine disturbance. Finnish male trained soldiers had increased GH and cortisol, and decreased testosterone and LH, following a 7 day field exercise in an energy deficit of ~4,000 kcal·d^−1^ (94). These markers of endocrine function recovered during the subsequent 2 weeks of field exercise when the energy deficit was decreased to < 1,000 kcal·d^−1^. Similarly, endocrine function was preserved in Norwegian special forces soldiers during 3 weeks of training with *ad libitum* food intake, but a subsequent 7 day field exercise in ~4,000 kcal·d^−1^ energy deficit increased cortisol and SHGB, and decreased IGF-1, T3, T4, and testosterone (95). The periods of most severe energy deficit likely coincide with periods of highest physical activity, sleep deprivation, and psychological stress. It is, therefore, not possible to identify the independent effects of energy status in these studies and randomized supplementation trials are required.

The few studies that have provided supplementary energy in addition to the habitual dietary intake during training provide further support for energy status as the mechanism for endocrine disturbances. An additional intake of 6,000–8,500 kcal·d^−1^ during the 5 day Norwegian Ranger course attenuated disturbances to GH, cortisol and thyroid hormones, but not reproductive hormones (74, 77, 78), whereas the provision of 3–6 h extra sleep per night had no protective effect (74, 76, 79). Increasing energy intake from 1,800 kcal·d^−1^ to 3,200 kcal·d^−1^ or 4,200 kcal·d^−1^ did not prevent the decrease in testosterone after a 5 day combat course, but *estimated* energy expenditure was > 5,000 kcal·d^−1^ and so the supplementary energy was insufficient to eliminate the energy deficit (96). Periods of re-feeding *throughout* US Army Ranger training resulted in the recovery of cortisol, IGF-1, T3, SHBG, and testosterone, but the provision of an extra 400 kcal·d^−1^ had no effect on these markers, which was also insufficient to eliminate the energy deficit (2). Supplementation with 1,218 kcal·d^−1^ during an 8 week British Army combat course did not prevent the increase in cortisol, or decrease in IGF-1 and testosterone compared with a control group, but only 66% of the supplement was consumed resulting in only a small attenuation of the energy deficit (4). Disturbances to adrenal, thyroid, reproductive, and metabolic hormones may depend on the severity of energy deficit, and some of these disturbances can be attenuated by additional energy intake, supporting a role of energy deficit, at least in part, in the endocrine disturbances observed with military activities.

### Menstrual Function

Chronic low energy availability can result in functional hypothalamic amenorrhoea in women. Functional hypothalamic amenorrhoea is the absence of menses due to suppression of the hypothalamic-pituitary-ovarian axis (97). The suppressed pulsatile release of gonadotropin-releasing hormone from the hypothalamus, decreases LH pulse frequency from the pituitary, and results in low circulating oestradiol (15, 16). Several studies have investigated menstrual disturbances in the military (24, 98–101), but inconsistencies with definitions of, and screening methods used for, menstrual disturbances, limit robust conclusions [see Gifford et al. (102) for a comprehensive review of menstrual dysfunction in the military]. Cross-sectional studies in the US Army identified the prevalence of menstrual disturbances was 11–15% (amenorrhoea, oligomenorrhoea, or delayed menarche) (24, 99). More than 90% of women reported menstrual irregularities during 1 year of basic military training at the US military academy, with almost half of the female cohort reporting decreased menstrual frequency (98, 100). A similarly high prevalence (70%) of menstrual disturbances were reported after starting Korean basic military training (92). Although these studies demonstrate basic military training can disturb menstrual function, the role of energy availability is not clear. Hormonal contraceptive use is also higher in servicewomen than their civilian counterparts, possibly due to an increased desire to suppress menses (103). A high use of hormonal contraception in servicewomen may mask changes in menstrual function in military training. To our knowledge, there are no prospective data examining the relationship between energy status and menstrual function in military women, and other factors such as psychological stress may contribute to menstrual function disturbances (92, 101, 102).

### Bone

Poor skeletal health is a widely recognized clinical outcome associated with chronic low energy availability, and forms part of the Triad (11) and RED-S (9). Low energy availability decreases bone formation, possibly mediated by increased cortisol and decreased T3, leptin, and IGF-1, and can increase bone resorption by decreasing oestradiol production in women (9, 11, 14, 102, 104). Amenorrhoeic athletes have lower whole-body, axial, and appendicular areal BMD (105–108), poorer bone microarchitecture and/or mechanical strength (105, 107, 109, 110), and increased stress fracture risk (105) compared with their eumenorrhoeic counterparts. Low energy availability and low oestradiol may have independent and combined effects on bone (110, 111). The relationship between low energy availability and bone in men is less well-described, but endocrine disturbances, including decreases in sex steroids and IGF-1, likely disturb bone health (27, 112, 113).

Male US Army Rangers had decreased markers of bone formation (bone-specific alkaline phosphatase) and increased markers of bone resorption (tartrate-resistant acid phosphatase) after 8 weeks of training with energy deficits of ~1,000 kcal·d^−1^ (114). Bone-specific alkaline phosphatase is indicative of bone mineralisation and tartrate-resistant acid phosphatase reflects osteoclast number (115), whereas C-telopeptide cross-links of type 1 collagen, a marker of type I collagen breakdown, was unchanged. Bone-specific alkaline phosphatase was still decreased 2–6 weeks after the cessation of training and when body mass had returned to pre-training values, whereas tartrate-resistant acid phosphatase had returned to baseline, indicating a lag in bone formation and a vulnerable period for overloading bone. This reduction in bone formation coincided with decreases in whole-body bone mineral content (2). Whole-body bone mineral content and areal BMD losses were also evident after 7–10 months of operational deployment, during which time a 1.9% loss in body mass was observed (116). Six female soldiers who lost 13% body mass over a 61 day crossing of the Antarctic had decreased bone formation and areal BMD at the axial skeleton (117). Tibial macro and microstructure were unchanged suggesting mechanical loading was protective during energy deficiency. These studies demonstrate that specialist military training and deployment in energy deficit results in decreased bone formation, increased bone resorption, and loss of bone mass from the axial skeleton.

Stress fractures at weight-bearing sites are common during basic military training (25, 118, 119), more so in women than in men (120, 121). Skeletal injuries are indicative of the high mechanical stresses of military activities, but decreased bone formation and increased bone resorption with low energy availability could decrease mechanical strength of bone and increase the propagation of microcracks with repeated loading. The evidence for low energy availability in basic military training is mixed, and bone adapts favorably in response to 8–13 weeks basic military training programmes at appendicular sites (122–126). Increased incidence of stress fractures (25, 99, 127–129) and musculoskeletal injuries (130), and lower bone mass (131), are seen in servicewomen with menstrual disturbances (oligomenorrhoea, amenorrhoea, or delayed menarche), although these findings are not supported by all studies (24, 132–136). Disordered eating (25, 134, 136) and self-reported dietary intake (133, 137) are also not predictive of stress fracture, but these studies must be interpreted with consideration for the limitations of measuring dietary behaviors. Studies have also demonstrated a greater decrease in body mass (136) and IGF-1 (138), indicative of energy deficiency, in stress fracture cases compared with non-injured controls during basic military training. Decreased bone formation, increased bone resorption, and bone loss from the axial skeleton are observed during specialist military training, field exercises, and operational deployment in military personnel experiencing energy deficit, but the link between energy deficit and stress fracture in this population is unclear.

### Immune Function

Cell-mediated and humoral immune function is disturbed by specialist military training in energy deficit (5 days to 8 weeks) (35, 139–149). High levels of physical activity, insufficient micronutrient intake, exposure to environmental extremes, sleep deprivation, and psychological stress may have contributed to these responses (150, 151), but providing an energy supplement attenuates some of these effects. Eight weeks of US Army Ranger training in energy deficit suppressed *in vitro* T-lymphocyte function and resulted in a high incidence of infection, which were both attenuated with an additional ~400 kcal·d^−1^ of energy intake (140, 152). The provision of an extra 1,218 kcal·d^−1^ during an 8 week British Army combat course maintained circulating leukocytes, lymphocytes, and monocytes, and increased the secretory rate of salivary immunoglobulin A compared with a control group (139). Similarly, increasing energy intake by providing fresh food rather than a ration pack maintained salivary immunoglobulin A during an Australian Air Force 12-day tropical field exercise (35). The clinical relevance of these acute changes in immune function is unclear, however, post-exercise protein supplementation throughout US Marine basic military training decreased the number of visits to the medical center for illness (153), providing some support for energy status in clinical outcomes. Basic military training (19–20 weeks) with high total energy expenditures and psychological stress, but stable body mass, had minimal effect on markers of immune function and the incidence of upper respiratory tract infection (154, 155), further supporting an independent role of energy status on immune function. These studies provide some support for a role of energy deficiency in disturbed immune function during military training.

### Gastrointestinal

Gastrointestinal distress is common during specialist military training (156) and operational deployment (157). Restricting the gut of essential nutrients places stress on the microbiota, and changes in gut microbiota may contribute to decrements in gut health during energy deficit (158). Increased intestinal permeability and marked changes in gut microbiota and gut microbiota derived metabolites were observed after 4 days of severe energy deficit in Norwegian soldiers (159). An increase in intestinal permeability is consistently reported following specialist military training and could contribute to the increase in gastrointestinal symptoms (156, 160). Changes to the gut microbiota could be important in the etiology of several other clinical outcomes including musculoskeletal injury, illness and infection, and psychological impairments (158), but the role of energy deficit on these outcomes is not clear. High levels of physical activity, inflammation, environmental extremes, sleep deprivation, psychological stress, and changes to diet composition could all contribute to these gastrointestinal changes (158).

### Hematological

Low energy availability may play a role in iron deficiency commonly seen in female athletes (10). Iron status is determined by the measurement of a combination of biochemical markers including ferritin, transferrin saturation, soluble transferrin receptor, and hemoglobin (161, 162). Widespread disturbances in these markers of iron status were reported in men and women following 7–26 weeks of basic military training (137, 163–169) and 12–26 weeks of operational deployment (39, 170). Stable or small losses in body mass in these studies, and similar self-reported habitual energy intake between recruits with and without iron deficiency (171), suggest energy deficit was not a primary mechanism in impaired iron status. Specialist military training in energy deficit does not affect iron status in trained male soldiers, although small decreases in hemoglobin have been observed (162, 172). Ferritin and hepcidin, a regulator of iron status, increased during specialist military training courses in energy deficit in trained male soldiers (57, 81, 162, 173, 174), indicative of increased inflammation rather than improved iron status (162). These studies suggest that changes in iron status with military training and operations are independent of energy deficits; increased physical activity and inflammation, decreased iron intake, gastrointestinal bleeding, iron sweat loss, and an increase in iron turnover are potential mechanisms (163, 166).

### Psychological

Numerous studies have reported mood disturbances, as measured by the Profile of Mood States, in response to specialist military training of several days to 8 weeks in energy deficit (35, 147, 173, 175–183). The 8 week US Army Ranger course increased tension, depression, anger, fatigue, and confusion, and decreased vigor (176); similar disturbances to mood have been reported following just 3 days of sustained military activities in severe energy deficit (~3,000 kcal·d^−1^) (175, 178). US basic military training improved mood for both men and women (166, 184–186), where energy intake was more likely matched to energy expenditure (124, 187). Conversely, women undergoing the 44-week British Army Officer basic military training course had decreased resilience and increased depression (93). Additional carbohydrate improved vigor and decreased confusion during a day of military activities, but other mood constructs were not affected and overall energy status was not measured (188). Supplementary energy during field exercises in an energy deficit had limited impact on mood disturbance (182, 189, 190), but the provision of fresh feeding rather than ration packs, which also resulted in increased energy intake, attenuated feelings of fatigue during a 12 day field exercise in energy deficit (35). Although controlled laboratory trials provide some support for a role of energy deficiency in mood disturbances (191, 192), psychological stress is a fundamental component of military training, and combined with sleep deprivation and the potential for dehydration, mood disturbances cannot be attributed solely to energy deficit.

## Energy Deficiency and Performance

### Physical Performance

Muscle strength and power is decreased following specialist military training courses of 3 days to 8 weeks in energy deficits of ~500–4,000 kcal·d^−1^ (4, 46, 48, 58, 80, 81, 89, 95, 175, 189, 193). Lower limb muscle power, assessed by jump performance, is decreased by 9–28% (4, 48, 58, 80, 81, 89). Not all studies report a decrease in lower limb muscle performance (189, 194, 195) and muscle fatigue and/or damage likely contributes to some of these effects (29, 46, 196). The maximal weight that can be lifted during whole-body exercise is decreased by 14–21% (4, 80, 81, 193) and upper body strength is decreased by up to 10% (48, 95, 189). An impairment in upper body strength and endurance is not consistently observed (35, 175, 194), but occupational task performance (repetitive lifting, obstacle course, and wall building) can still decrease despite maintained upper body strength (175), demonstrating the importance of testing relevant aspects of military occupational performance. It is not possible to identify the direct role of energy deficit on impaired muscle performance from these studies, and military activities will result in some exercise-induced muscle fatigue and damage. Several days of fasting decreased upper body strength in male soldiers in the absence of other stressors (197), providing support for an independent effect of energy deficiency on muscle performance. A meta-regression demonstrated that muscle performance is impaired in proportion to the energy deficit, and limiting energy deficits to absolute values of −5,686 to −19,109 kcal, or ≤ 3% of body mass, for an entire operation will limit muscle performance decrements to ≤ 2% (198).

There are fewer data on endurance performance parameters. Decreased aerobic performance is seen after several days of field exercise in energy deficit (96, 181) but anaerobic performance is generally protected (96). Longer periods of operational deployment (6–13 months) result in unchanged (39, 199) or decreased (5–13%) (116, 200) aerobic capacity, and unchanged or increased muscle performance (39, 87, 116, 199). Modest decreases, or even increases in body mass, following deployment suggests the energy deficit was not severe in these studies. The delay between the end of deployment and the measurement of physical performance, the potential for de-conditioning (116, 200) or increased physical training volume (199), differences in operational role performed (199), and the temporal nature of combat activity, make studies on deployments difficult to interpret.

The provision of an additional 1,218 kcal·d^−1^ during an 8 week British Army combat course attenuated the energy deficit from ~500 kcal·d^−1^ to 150 kcal·d^−1^ and prevented the loss in FFM and muscle performance (4). Another study found no effect of supplementary energy on physical performance and FFM, although the energy failed to offset the energy deficit compared with a control group (189). Muscle performance decreases in proportion to the loss in FFM (4, 80, 193), with energy deficit resulting in negative whole-body protein balance (47, 201). Other energy supplementation studies demonstrated that aerobic performance loss is attenuated following a 5 day field exercise (96), and performance on military physical fitness tests is augmented (202) and musculoskeletal injury incidence is decreased (153, 203) in basic military training. These studies support energy deficiency as a contributing factor in impaired muscle performance, and to a lesser extent aerobic endurance, during military training.

### Cognitive Performance

Impaired vigilance, choice and simple reaction time, pattern recognition, short-term working memory, logical reasoning, and marksmanship are reported following several days to 8 weeks of specialist military training in energy deficit (173, 176–180, 182, 204), although other studies report unchanged (35) or improved (192) cognitive performance. Although impaired cognitive performance coincided with severe energy deficits (173, 176, 178), and demonstrated recovery with re-feeding (176), laboratory studies demonstrated that 2 days of isolated energy deficit only decreases cognitive performance during exercise (205) and not at rest (191, 206). In contrast, components of logical reasoning, working memory, visual reaction time, and vigilance improved in US Army basic military training (185). Additional energy in the form of carbohydrate supplementation maintained vigilance during 1 day of sustained simulated military activity compared with a placebo (188), and promoted recovery of Stroop test performance following special forces survival training (207), although energy status was unreported. Shooting performance decreased during a 21 day field exercise, and although energy balance was not measured, decreased IGF-1 suggests an energy deficit (194). Shorter periods (4 days) of field training in severe energy deficit (~2,900 kcal·d^−1^) had no effect on shooting performance (175) and high energy intakes did not improve shooting performance (208) or protect against decrements in other constructs of cognitive performance (182, 209) during field exercises in energy deficit. The multi-stressors of military training, including sleep deprivation and dehydration, may contribute to impaired cognitive performance, with sleep deprivation and energy restriction likely to have independent effects (210).

## Energy Deficiency in Female Soldiers

Most evidence for the effect of low energy availability on health and performance is in female athletes (9–11, 19), whereas most of the military studies presented here were conducted in men. The effect of low energy availability in male athletes is being increasingly recognized (27, 112), and these military data make an important contribution to this field. The lack of data on military women is likely due, in part, to their previous exclusion from combat roles, and, therefore, lack of exposure to severe energy deficits. Men make-up most of the military, and women have only recently been permitted to enter combat roles in several countries including the UK and US. Women in these combat roles are likely to experience higher physical demands (29, 196, 211), have poorer physical performance (211), a higher incidence of musculoskeletal injuries and stress fractures (121), and increased risk of reproductive disturbances with low energy availability (102), compared with men. Better understanding of the effects of low energy availability on the health and performance of women in military roles, including combat roles, is an important area of future study.

## Conclusions

**Figure 1:**
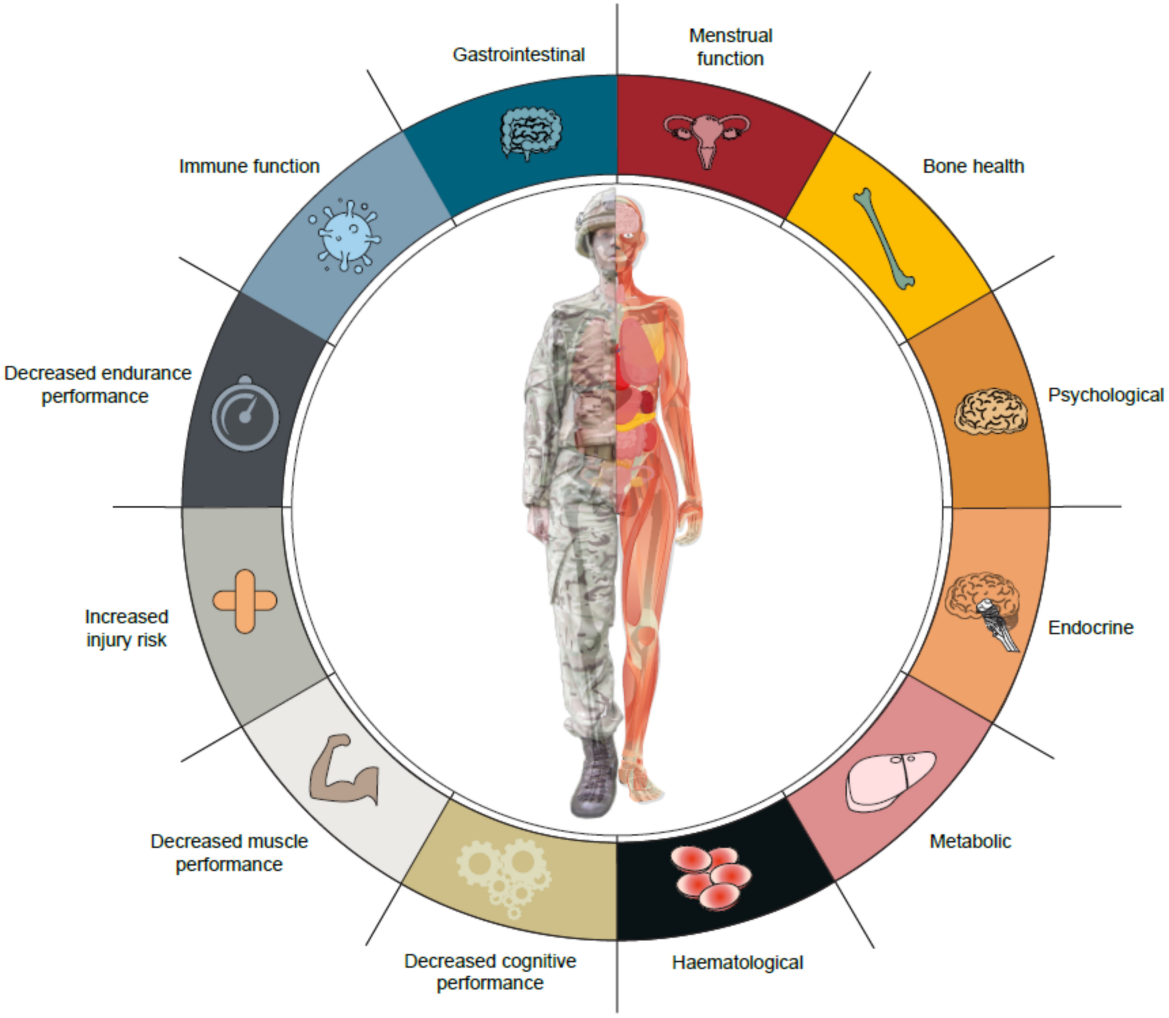
Potential health and performance effects of military training and operations in energy deficit.

Military personnel are exposed to energy deficits of varying severities during training and on operational deployment. These energy deficits are largely experienced by trained soldiers during specialist military combat training courses and field exercises, rather than recruits in basic military training. Military training in energy deficit results in many components of the Triad and RED-S, notably disturbances to endocrine and metabolic function, bone turnover, immune function, gastrointestinal health, mood, and physical and cognitive performance (Figure 1). Military training is a multi-stressor environment, so it is difficult to isolate the independent effects of energy status. Energy supplementation studies suggest that energy deficiency contributes to impaired metabolic, endocrine, and immune function, and physical performance. Further randomized controlled trials are required to better identify the role of feeding and energy deficiency on health and performance outcomes. Most studies examined the short-term effects of arduous military training courses, but the long-term health effects of cyclical phases of energy deficiency and recovery, characteristic of military training and employment, require further study. More prospective longitudinal studies are also important to better understand the effect of energy deficiency on female soldiers' health and performance.

## Author Contributions

TO'L performed the literature search and produced the manuscript. SW and JG edited the manuscript for intellectual content. All authors made contributions to the conception of the review and approved the final manuscript.

## Conflict of Interest

The authors declare that the research was conducted in the absence of any commercial or financial relationships that could be construed as a potential conflict of interest.

## References

[1] Richmond VL Horner FE Wilkinson DM Rayson MP Wright A Izard R. Energy balance and physical demands during an 8-week arduous military training course. Mil Med. (2014) 179:421–7. 10.7205/MILMED-D-13-00313 24690967

[2] Friedl KE Moore RJ Hoyt RW Marchitelli LJ Martinez-Lopez LE Askew EW. Endocrine markers of semistarvation in healthy lean men in a multistressor environment. J Appl Physiol. (2000) 88:1820–30. 10.1152/jappl.2000.88.5.1820 10797147

[3] Margolis LM Rood J Champagne C Young AJ Castellani JW. Energy balance and body composition during US Army special forces training. Appl Physiol Nutr Metabol. (2013) 38:396–400. 10.1139/apnm-2012-0323 23713532

[4] Fortes MB Diment BC Greeves JP Casey A Izard R Walsh NP. Effects of a daily mixed nutritional supplement on physical performance, body composition, and circulating anabolic hormones during 8 weeks of arduous military training. Appl Physiol Nutr Metabol. (2011) 36:967–75. 10.1139/h11-124 22111592

[5] Hoyt RW Friedl KE. Field studies of exercise and food deprivation. Curr Opin Clin Nutr Metab Care. (2006) 9:685–90. 10.1097/01.mco.0000247472.72155.7c 17053420

[6] Tharion WJ Lieberman HR Montain SJ Young AJ Baker-Fulco CJ Delany JP. Energy requirements of military personnel. Appetite. (2005) 44:47–65. 10.1016/j.appet.2003.11.010 15604033

[7] Tassone EC Baker BA. Body weight and body composition changes during military training and deployment involving the use of combat rations: a systematic literature review. Br J Nutr. (2017) 117:897–910. 10.1017/S0007114517000630 28452292

[8] Barringer ND Pasiakos SM McClung HL Crombie AP Margolis LM. Prediction equation for estimating total daily energy requirements of special operations personnel. J Int Soc Sports Nutr. (2018) 15:15. 10.1186/s12970-018-0219-x 29632452 PMC5885383

[9] Mountjoy M Sundgot-Borgen J Burke L Carter S Constantini N Lebrun C. The IOC consensus statement: beyond the female athlete triad–relative energy deficiency in sport (RED-S). Br J Sports Med. (2014) 48:491–7. 10.1136/bjsports-2014-093502 24620037

[10] Mountjoy M Sundgot-Borgen J Burke L Ackerman KE Blauwet C Constantini N. International Olympic Committee (IOC) consensus statement on relative energy deficiency in sport (RED-S): 2018 update. Int J Sport Nutr Exerc Metabol. (2018) 28:316–31. 10.1123/ijsnem.2018-0136 29771168

[11] Nattiv A Loucks AB Manore MM Sanborn CF Sundgot-Borgen J Warren MP. American college of sports medicine position stand. The female athlete triad. Med Sci Sports Exerc. (2007) 39:1867–82. 10.1249/mss.0b013e318149f111 17909417

[12] Loucks AB Kiens B Wright HH. Energy availability in athletes. J Sports Sci. (2011) 29(Suppl. 1):S7–15. 10.1080/02640414.2011.588958 21793767

[13] Wade GN Jones JE. Neuroendocrinology of nutritional infertility. Am J Physiol Regul Integr Comp Physiol. (2004) 287:R1277–96. 10.1152/ajpregu.00475.2004 15528398

[14] Ihle R Loucks AB. Dose-response relationships between energy availability and bone turnover in young exercising women. J Bone Miner Res. (2004) 19:1231–40. 10.1359/JBMR.040410 15231009

[15] Loucks AB Thuma JR. Luteinizing hormone pulsatility is disrupted at a threshold of energy availability in regularly menstruating women. J Clin Endocrinol Metabol. (2003) 88:297–311. 10.1210/jc.2002-020369 12519869

[16] Loucks AB Verdun M Heath EM. Low energy availability, not stress of exercise, alters LH pulsatility in exercising women. J Appl Physiol. (1998) 84:37–46. 10.1152/jappl.1998.84.1.37 9451615

[17] Loucks AB Heath EM. Induction of low-T3 syndrome in exercising women occurs at a threshold of energy availability. Am J Physiol. (1994) 266(3 Pt 2):R817–23. 10.1152/ajpregu.1994.266.3.R817 8160876

[18] Loucks AB. Energy balance and body composition in sports and exercise. J Sports Sci. (2004) 22:1–14. 10.1080/0264041031000140518 14974441

[19] De Souza MJ Nattiv A Joy E Misra M Williams NI Mallinson RJ. 2014 Female Athlete Triad Coalition consensus statement on treatment and return to play of the female athlete triad: 1st International Conference held in San Francisco, California, may 2012 and 2nd International Conference held in Indianapolis, Indiana, May 2013. Br J Sports Med. (2014) 48:289. 10.1136/bjsports-2013-093218 24463911

[20] Otis CL Drinkwater B Johnson M Loucks A Wilmore J. American college of sports medicine position stand. The female athlete triad. Med Sci Sports Exerc. (1997) 29:i–ix. 10.1097/00005768-199705000-00037 9140913

[21] Mountjoy M Sundgot-Borgen J Burke L Carter S Constantini N Lebrun C. Authors' 2015 additions to the IOC consensus statement: relative energy deficiency in sport (RED-S). Br J Sports Med. (2015) 49:417–20. 10.1136/bjsports-2014-094371 25950026

[22] Williams NI Koltun KJ Strock NCA De Souza MJ. Female athlete triad and relative energy deficiency in sport: a focus on scientific rigor. Exerc Sport Sci Rev. (2019) 47:197–205. 10.1249/JES.0000000000000200 31524785

[23] Friedl KE. Body composition and military performance–many things to many people. J Strength Cond Res. (2012) 26(Suppl. 2):S87–100. 10.1519/JSC.0b013e31825ced6c 22643136

[24] Lauder TD Williams MV Campbell CS Davis G Sherman R Pulos E. The female athlete triad: prevalence in military women. Mil Med. (1999) 164:630–5. 10.1093/milmed/164.9.630 10495633

[25] Cosman F Ruffing J Zion M Uhorchak J Ralston S Tendy S. Determinants of stress fracture risk in United States military academy cadets. Bone. (2013) 55:359–66. 10.1016/j.bone.2013.04.011 23624291

[26] Torstveit MK Sundgot-Borgen J. The female athlete triad exists in both elite athletes and controls. Med Sci Sports Exerc. (2005) 37:1449–59. 10.1249/01.mss.0000177678.73041.38 16177594

[27] Tenforde AS Barrack MT Nattiv A Fredericson M. Parallels with the female athlete triad in male athletes. Sports Med. (2016) 46:171–82. 10.1007/s40279-015-0411-y 26497148

[28] Nindl BC Jones BH Van Arsdale SJ Kelly K Kraemer WJ. Operational physical performance and fitness in military women: physiological, musculoskeletal injury, and optimized physical training considerations for successfully integrating women into combat-centric military occupations. Mil Med. (2016) 181(Suppl. 1):50–62. 10.7205/MILMED-D-15-00382 26741902

[29] O'Leary TJ Saunders SC McGuire SJ Venables MC Izard RM. Sex differences in training loads during British army basic training. Med Sci Sports Exerc. (2018) 50:2565–74. 10.1249/MSS.0000000000001716 30048410

[30] Wilkinson DM Rayson MP Bilzon JL. A physical demands analysis of the 24-week British army parachute regiment recruit training syllabus. Ergonomics. (2008) 51:649–62. 10.1080/00140130701757367 18432443

[31] Blacker SD Wilkinson DM Rayson MP. Gender differences in the physical demands of British army recruit training. Mil Med. (2009) 174:811–6. 10.7205/MILMED-D-01-3708 19743735

[32] Richmond VL Carter JM Wilkinson DM Homer FE Rayson MP Wright A. Comparison of the physical demands of single-sex training for male and female recruits in the British army. Mil Med. (2012) 177:709–15. 10.7205/MILMED-D-11-00416 22730848

[33] Friedl KE Moore RJ Martinez-Lopez LE Vogel JA Askew EW Marchitelli LJ. Lower limit of body fat in healthy active men. J Appl Physiol. (1994) 77:933–40. 10.1152/jappl.1994.77.2.933 8002550

[34] Burke LM Lundy B Fahrenholtz IL Melin AK. Pitfalls of conducting and interpreting estimates of energy availability in free-living athletes. Int J Sport Nutr Exerc Metabol. (2018) 28:350–63. 10.1123/ijsnem.2018-0142 30029584

[35] Booth CK Coad RA Forbes-Ewan CH Thomson GF Niro PJ. The physiological and psychological effects of combat ration feeding during a 12-day training exercise in the tropics. Mil Med. (2003) 168:63–70. 10.1093/miled.168.1.63 12546249

[36] Forbes-Ewan CH Morrissey BL Gregg GC Waters DR. Use of doubly labeled water technique in soldiers training for jungle warfare. J Appl Physiol. (1989) 67:14–8. 10.1152/jappl.1989.67.1.14 2759938

[37] Carter J Wilkinson D Blacker S Rayson M Bilzon J Izard R. An investigation of a novel three-dimensional activity monitor to predict free-living energy expenditure. J Sports Sci. (2008) 26:553–61. 10.1080/02640410701708979 18344125

[38] Siddall AG Powell SD Needham-Beck SC Edwards VC Thompson JES Kefyalew SS. Validity of energy expenditure estimation methods during 10 days of military training. Scand J Med Sci Sports. (2019) 29:1313–21. 10.1111/sms.13488 31136027

[39] Fallowfield JL Delves SK Hill NE Cobley R Brown P Lanham-New SA. Energy expenditure, nutritional status, body composition and physical fitness of Royal Marines during a 6-month operational deployment in Afghanistan. Br J Nutr. (2014) 112:821–9. 10.1017/S0007114514001524 25007417

[40] Jones PJ Jacobs I Morris A Ducharme MB. Adequacy of food rations in soldiers during an arctic exercise measured by doubly labeled water. J Appl Physiol. (1993) 75:1790–7. 10.1152/jappl.1993.75.4.1790 8282633

[41] Tanskanen M Uusitalo AL Hakkinen K Nissila J Santtila M Westerterp KR. Aerobic fitness, energy balance, and body mass index are associated with training load assessed by activity energy expenditure. Scand J Med Sci Sports. (2009) 19:871–8. 10.1111/j.1600-0838.2008.00857.x 18980607

[42] Kinnunen H Tanskanen M Kyrolainen H Westerterp KR. Wrist-worn accelerometers in assessment of energy expenditure during intensive training. Physiol Meas. (2012) 33:1841–54. 10.1088/0967-3334/33/11/1841 23110981

[43] Blacker SD Horner FL Brown PI Linnane DM Wilkinson DM Wright A. Health, fitness, and responses to military training of officer cadets in a Gulf Cooperation Council country. Mil Med. (2011) 176:1376–81. 10.7205/MILMED-D-11-00166 22338351

[44] Burstein R Coward AW Askew WE Carmel K Irving C Shpilberg O. Energy expenditure variations in soldiers performing military activities under cold and hot climate conditions. Mil Med. (1996) 161:750–4. 10.1093/milmed/161.12.750 8990835

[45] Rietjens G Most J Joris PJ Helmhout P Plasqui G. Energy expenditure and changes in body composition during submarine deployment-an observational study “DasBoost 2–2017”. Nutrients. (2020) 12:226. 10.3390/nu12010226 31952273 PMC7019715

[46] Margolis LM Murphy NE Martini S Spitz MG Thrane I McGraw SM. Effects of winter military training on energy balance, whole-body protein balance, muscle damage, soreness, and physical performance. Appl Physiol Nutr Metabol. (2014) 39:1395–401. 10.1139/apnm-2014-0212 25386980

[47] Margolis LM Murphy NE Martini S Gundersen Y Castellani JW Karl JP. Effects of supplemental energy on protein balance during 4-d arctic military training. Med Sci Sports Exerc. (2016) 48:1604–12. 10.1249/MSS.0000000000000944 27054679

[48] Ofsteng SJ Garthe I Josok O Knox S Helkala K Knox B. No effect of increasing protein intake during military exercise with severe energy deficit on body composition and performance. Scand J Med Sci Sports. (2020) 30:865–77. 10.1111/sms.13634 32034812

[49] Hoyt RW Opstad PK Haugen AH DeLany JP Cymerman A Friedl KE. Negative energy balance in male and female rangers: effects of 7 d of sustained exercise and food deprivation. Am J Clin Nutr. (2006) 83:1068–75. 10.1093/ajcn/83.5.1068 16685048

[50] Proctor SP Scarpaci MM Maule AL Heaton KJ Taylor K Haven CC. Role of body composition and physical activity on permethrin urinary biomarker concentrations while wearing treated military uniforms. Toxicol Lett. (2018) 299:210–7. 10.1016/j.toxlet.2018.10.001 30292884 PMC7976610

[51] Edwards JSA Askew WE King N Fulco CS Nutritional intake and carbohydrate supplementation at high altitude. J Wilderness Med. (1994) 5:20–33. 10.1580/0953-9859-5.1.20

[52] McClung HL Champagne CM Allen HR McGraw SM Young AJ Montain SJ. Digital food photography technology improves efficiency and feasibility of dietary intake assessments in large populations eating *ad libitum* in collective dining facilities. Appetite. (2017) 116:389–94. 10.1016/j.appet.2017.05.025 28527951

[53] Nindl BC Friedl KE Marchitelli LJ Shippee RL Thomas CD Patton JF. Regional fat placement in physically fit males and changes with weight loss. Med Sci Sports Exerc. (1996) 28:786–93. 10.1097/00005768-199607000-00003 8832530

[54] Hoyt RW Jones TE Stein TP McAninch GW Lieberman HR Askew EW. Doubly labeled water measurement of human energy expenditure during strenuous exercise. J Appl Physiol. (1991) 71:16–22. 10.1152/jappl.1991.71.1.16 1917738

[55] Tharion WJ Hoyt RW Cline AD DeLany JP Lieberman HR Energy expenditure and water turnover assessed by doubly labeled water during manual work in a dry and warm environment. J Hum Environ Syst. (2004) 7:11–7. 10.1618/jhes.7.11

[56] Hoyt RW Buller MJ Santee WR Yokota M Weyand PG Delany JP. Total energy expenditure estimated using foot-ground contact pedometry. Diabetes Technol Ther. (2004) 6:71–81. 10.1089/152091504322783459 15000774

[57] Nindl BC Alemany JA Kellogg MD Rood J Allison SA Young AJ. Utility of circulating IGF-I as a biomarker for assessing body composition changes in men during periods of high physical activity superimposed upon energy and sleep restriction. J Appl Physiol. (2007) 103:340–6. 10.1152/japplphysiol.01321.2006 17412783

[58] Welsh TT Alemany JA Montain SJ Frykman PN Tuckow AP Young AJ. Effects of intensified military field training on jumping performance. Int J Sports Med. (2008) 29:45–52. 10.1055/s-2007-964970 17879876

[59] Alemany JA Nindl BC Kellogg MD Tharion WJ Young AJ Montain SJ. Effects of dietary protein content on IGF-I, testosterone, and body composition during 8 days of severe energy deficit and arduous physical activity. J Appl Physiol. (2008) 105:58–64. 10.1152/japplphysiol.00005.2008 18450989

[60] Sepowitz JJ Armstrong NJ Pasiakos SM. Energy balance and diet quality during the US marine corps forces special operations command individual training course. J Spec Oper Med. (2017) 17:109–13. 29256207 10.55460/RKM3-KDFU

[61] Berryman CE Sepowitz JJ McClung HL Lieberman HR Farina EK McClung JP. Supplementing an energy adequate, higher protein diet with protein does not enhance fat-free mass restoration after short-term severe negative energy balance. J Appl Physiol. (2017) 122:1485–93. 10.1152/japplphysiol.01039.2016 28385919

[62] Castellani JW Delany JP O'Brien C Hoyt RW Santee WR Young AJ. Energy expenditure in men and women during 54 h of exercise and caloric deprivation. Med Sci Sports Exerc. (2006) 38:894–900. 10.1249/01.mss.0000218122.59968.eb 16672843

[63] McClung HL Sigrist LD Smith TJ Karl JP Rood JC Young AJ. Monitoring energy intake: a hand-held personal digital assistant provides accuracy comparable to written records. J Am Diet Assoc. (2009) 109:1241–5. 10.1016/j.jada.2009.04.015 19559143

[64] Tharion WJ Yokota M Buller MJ DeLany JP Hoyt RW. Total energy expenditure estimated using a foot-contact pedometer. Med Sci Monit. (2004) 10:CR504–9. 15328482

[65] DeLany JP Schoeller DA Hoyt RW Askew EW Sharp MA. Field use of D2 18O to measure energy expenditure of soldiers at different energy intakes. J Appl Physiol. (1989) 67:1922–9. 10.1152/jappl.1989.67.5.1922 2600025

[66] Johnson CD Simonson AJ Darnell ME DeLany JP Wohleber MF Connaboy C. Energy expenditure and intake during special operations forces field training in a jungle and glacial environment. Appl Physiol Nutr Metabol. (2018) 43:381–6. 10.1139/apnm-2017-0622 29144888

[67] Margolis LM Crombie AP McClung HL McGraw SM Rood JC Montain SJ. Energy requirements of US army special operation forces during military training. Nutrients. (2014) 6:1945–55. 10.3390/nu6051945 24824290 PMC4042567

[68] Hoyt RW Jones TE Baker-Fulco CJ Schoeller DA Schoene RB Schwartz RS. Doubly labeled water measurement of human energy expenditure during exercise at high altitude. Am J Physiol. (1994) 266(3 Pt 2):R966–71. 10.1152/ajpregu.1994.266.3.R966 8160893

[69] Tharion WJ Baker-Fulco CJ Bovill ME Montain SM DeLany JP Champagne CM. Adequacy of garrison feeding for special forces soldiers during training. Mil Med. (2004) 169:483–90. 10.7205/MILMED.169.6.483 15281681

[70] Mudambo KS Scrimgeour CM Rennie MJ. Adequacy of food rations in soldiers during exercise in hot, day-time conditions assessed by doubly labelled water and energy balance methods. Eur J Appl Physiol. (1997) 76:346–51. 10.1007/s004210050259 9349650

[71] Pasiakos SM Margolis LM. Negative energy balance and loss of body mass and fat-free mass in military personnel subsisting on combat rations during training and combat operations: a comment on Tassone and Baker. Br J Nutr. (2017) 117:894–6. 10.1017/S0007114517000605 28443530

[72] Beals K Darnell ME Lovalekar M Baker RA Nagai T San-Adams T. Suboptimal nutritional characteristics in male and female soldiers compared to sports nutrition guidelines. Mil Med. (2015) 180:1239–46. 10.7205/MILMED-D-14-00515 26633668

[73] Chapman S Roberts J Smith L Rawcliffe A Izard R. Sex differences in dietary intake in British Army recruits undergoing phase one training. J Int Soc Sports Nutr. (2019) 16:59. 10.1186/s12970-019-0327-2 31823790 PMC6905050

[74] Opstad PK Falch D Oktedalen O Fonnum F Wergeland R. The thyroid function in young men during prolonged exercise and the effect of energy and sleep deprivation. Clin Endocrinol. (1984) 20:657–69. 10.1111/j.1365-2265.1984.tb00116.x 6432374

[75] Aakvaag A Bentdal Ø Quigstad K Walstad P Rønningen H Fonnum F Testosterone and testosterone binding globulin (TeBG) in young men during prolonged stress. Int J Androl. (1978) 1:22–31. 10.1111/j.1365-2605.1978.tb00573.x

[76] Aakvaag A Sand T Opstad PK Fonnum F. Hormonal changes in serum in young men during prolonged physical strain. Eur J Appl Physiol. (1978) 39:283–91. 10.1007/BF00421452 710393

[77] Opstad PK Aakvaag A. The effect of a high calory diet on hormonal changes in young men during prolonged physical strain and sleep deprivation. Eur J Appl Physiol. (1981) 46:31–9. 10.1007/BF00422172 7014216

[78] Opstad PK Aakvaag A. Decreased serum levels of oestradiol, testosterone and prolactin during prolonged physical strain and sleep deprivation, and the influence of a high calorie diet. Eur J Appl Physiol. (1982) 49:343–8. 10.1007/BF00441295 6890449

[79] Opstad PK Aakvaag A. The effect of sleep deprivation on the plasma levels of hormones during prolonged physical strain and calorie deficiency. Eur J Appl Physiol. (1983) 51:97–107. 10.1007/BF00952542 6684038

[80] Nindl BC Barnes BR Alemany JA Frykman PN Shippee RL Friedl KE. Physiological consequences of U.S. army ranger training. Med Sci Sports Exerc. (2007) 39:1380–7. 10.1249/MSS.0b013e318067e2f7 17762372

[81] Nindl BC Friedl KE Frykman PN Marchitelli LJ Shippee RL Patton JF. Physical performance and metabolic recovery among lean, healthy men following a prolonged energy deficit. Int J Sports Med. (1997) 18:317–24. 10.1055/s-2007-972640 9298770

[82] Henning PC Scofield DE Spiering BA Staab JS Matheny RW Jr Smith MA. Recovery of endocrine and inflammatory mediators following an extended energy deficit. J Clin Endocrinol Metabol. (2014) 99:956–64. 10.1210/jc.2013-3046 24423293

[83] Gomez-Merino D Chennaoui M Drogou C Guezennec CY. Influence of energy deficiency on the insulin-like growth factor I axis in a military training program. Horm Metab Res. (2004) 36:506–11. 10.1055/s-2004-825730 15305236

[84] Nindl BC Rarick KR Castellani JW Tuckow AP Patton JF Young AJ. Altered secretion of growth hormone and luteinizing hormone after 84 h of sustained physical exertion superimposed on caloric and sleep restriction. J Appl Physiol. (2006) 100:120–8. 10.1152/japplphysiol.01415.2004 16141374

[85] Nindl BC Castellani JW Young AJ Patton JF Khosravi MJ Diamandi A. Differential responses of IGF-I molecular complexes to military operational field training. J Appl Physiol. (2003) 95:1083–9. 10.1152/japplphysiol.01148.2002 12909598

[86] Hill NE Woods DR Delves SK Murphy KG Davison AS Brett SJ. The gonadotrophic response of Royal Marines during an operational deployment in Afghanistan. Andrology. (2015) 3:293–7. 10.1111/andr.308 25645013

[87] Farina EK Taylor JC Means GE Williams KW Murphy NE Margolis LM. Effects of combat deployment on anthropometrics and physiological status of U.S. army special operations forces soldiers. Mil Med. (2017) 182:e1659–68. 10.7205/MILMED-D-16-00022 28290940

[88] Jensen AE Arrington LJ Turcotte LP Kelly KR. Hormonal balance and nutritional intake in elite tactical athletes. Steroids. (2019) 152:108504. 10.1016/j.steroids.2019.108504 31586604

[89] Vikmoen O Teien HK Raustol M Aandstad A Tanso R Gulliksrud K. Sex differences in the physiological response to a demanding military field exercise. Scand J Med Sci Sports. (2020) 1–12. 10.1111/sms.13689. [Epub ahead of print]. 32311789

[90] Gifford RM O'Leary T Cobb R Blackadder-Weinstein J Double R Wardle SL. Female reproductive, adrenal, and metabolic changes during an antarctic traverse. Med Sci Sports Exerc. (2019) 51:556–67. 10.1249/MSS.0000000000001803 30308528

[91] Hattersley J Wilson AJ Gifford RM Cobb R Thake CD Reynolds RM. Pre- to postexpedition changes in the energy usage of women undertaking sustained expeditionary polar travel. J Appl Physiol. (2019) 126:681–90. 10.1152/japplphysiol.00792.2018 30571278

[92] Cho GJ Han SW Shin JH Kim T. Effects of intensive training on menstrual function and certain serum hormones and peptides related to the female reproductive system. Medicine. (2017) 96:e6876. 10.1097/MD.0000000000006876 28538378 PMC5457858

[93] Gifford RM O'Leary TJ Double RL Wardle SL Wilson K Boyle LD. Positive adaptation of HPA axis function in women during 44 weeks of infantry-based military training. Psychoneuroendocrinology. (2019) 110:104432. 10.1016/j.psyneuen.2019.104432 31536944

[94] Kyrolainen H Karinkanta J Santtila M Koski H Mantysaari M Pullinen T. Hormonal responses during a prolonged military field exercise with variable exercise intensity. Eur J Appl Physiol. (2008) 102:539–46. 10.1007/s00421-007-0619-0 18040709

[95] Hamarsland H Paulsen G Solberg PA Slaathaug OG Raastad T. Depressed physical performance outlasts hormonal disturbances after military training. Med Sci Sports Exerc. (2018) 50:2076–84. 10.1249/MSS.0000000000001681 29927875

[96] Guezennec CY Satabin P Legrand H Bigard AX. Physical performance and metabolic changes induced by combined prolonged exercise and different energy intakes in humans. Eur J Appl Physiol. (1994) 68:525–30. 10.1007/BF00599524 7957146

[97] Gordon CM. Clinical practice. Functional hypothalamic amenorrhea. N Engl J Med. (2010) 363:365–71. 10.1056/NEJMcp0912024 20660404

[98] Schneider MB Fisher M Friedman SB Bijur PE Toffler AP. Menstrual and premenstrual issues in female military cadets: a unique population with significant concerns. J Pediatr Adolesc Gynecol. (1999) 12:195–201. 10.1016/S1083-3188(99)00025-X 10584223

[99] Friedl KE Nuovo JA Patience TH Dettori JR. Factors associated with stress fracture in young army women: indications for further research. Mil Med. (1992) 157:334–8. 10.1093/milmed/157.7.334 1528465

[100] Schneider MB Bijur PE Fisher M Friedman SB Toffler CP. Menstrual irregularity in female military cadets: comparison of data utilizing short-term and long-term recall. J Pediatr Adolesc Gynecol. (2003) 16:89–93. 10.1016/S1083-3188(03)00008-1 12742143

[101] Gordley LB Lemasters G Simpson SR Yiin JH. Menstrual disorders and occupational, stress, and racial factors among military personnel. J Occup Environ Med. (2000) 42:871–81. 10.1097/00043764-200009000-00005 10998762

[102] Gifford RM Reynolds RM Greeves J Anderson RA Woods DR. Reproductive dysfunction and associated pathology in women undergoing military training. J R Army Med Corps. (2017) 163:301–10. 10.1136/jramc-2016-000727 28213614

[103] Holt K Grindlay K Taskier M Grossman D. Unintended pregnancy and contraceptive use among women in the U.S. military: a systematic literature review. Mil Med. (2011) 176:1056–64. 10.7205/MILMED-D-11-00012 21987966

[104] Papageorgiou M Dolan E Elliott-Sale KJ Sale C. Reduced energy availability: implications for bone health in physically active populations. Eur J Nutr. (2018) 57:847–59. 10.1007/s00394-017-1498-8 28721562 PMC5861178

[105] Ackerman KE Cano Sokoloff N De Nardo Maffazioli G Clarke HM Lee H Misra M. Fractures in relation to menstrual status and bone parameters in young athletes. Med Sci Sports Exerc. (2015) 47:1577–86. 10.1249/MSS.0000000000000574 25397605 PMC4430468

[106] Cobb KL Bachrach LK Greendale G Marcus R Neer RM Nieves J. Disordered eating, menstrual irregularity, and bone mineral density in female runners. Med Sci Sports Exerc. (2003) 35:711–9. 10.1249/01.MSS.0000064935.68277.E7 12750578

[107] Ackerman KE Nazem T Chapko D Russell M Mendes N Taylor AP. Bone microarchitecture is impaired in adolescent amenorrheic athletes compared with eumenorrheic athletes and nonathletic controls. J Clin Endocrinol Metabol. (2011) 96:3123–33. 10.1210/jc.2011-1614 21816790 PMC3200253

[108] Piasecki J Ireland A Piasecki M Cameron J McPhee JS Degens H. The strength of weight-bearing bones is similar in amenorrheic and eumenorrheic elite long-distance runners. Scand J Med Sci Sports. (2018) 28:1559–68. 10.1111/sms.13062 29380429

[109] Ackerman KE Putman M Guereca G Taylor AP Pierce L Herzog DB. Cortical microstructure and estimated bone strength in young amenorrheic athletes, eumenorrheic athletes and non-athletes. Bone. (2012) 51:680–7. 10.1016/j.bone.2012.07.019 22878154 PMC3482939

[110] Southmayd EA Mallinson RJ Williams NI Mallinson DJ De Souza MJ. Unique effects of energy versus estrogen deficiency on multiple components of bone strength in exercising women. Osteoporos Int. (2017) 28:1365–76. 10.1007/s00198-016-3887-x 28032184

[111] De Souza MJ West SL Jamal SA Hawker GA Gundberg CM Williams NI. The presence of both an energy deficiency and estrogen deficiency exacerbate alterations of bone metabolism in exercising women. Bone. (2008) 43:140–8. 10.1016/j.bone.2008.03.013 18486582

[112] Burke LM Close GL Lundy B Mooses M Morton JP Tenforde AS. Relative energy deficiency in sport in male athletes: a commentary on its presentation among selected groups of male athletes. Int J Sport Nutr Exerc Metabol. (2018) 28:364–74. 10.1123/ijsnem.2018-0182 30040508

[113] Zanker CL Swaine IL. Responses of bone turnover markers to repeated endurance running in humans under conditions of energy balance or energy restriction. Eur J Appl Physiol. (2000) 83:434–40. 10.1007/s004210000293 11138586

[114] Hughes JM Smith MA Henning PC Scofield DE Spiering BA Staab JS. Bone formation is suppressed with multi-stressor military training. Eur J Appl Physiol. (2014) 114:2251–9. 10.1007/s00421-014-2950-6 25027064

[115] Dolan E Varley I Ackerman KE Pereira RMR Elliott-Sale KJ Sale C. The bone metabolic response to exercise and nutrition. Exerc Sport Sci Rev. (2020) 48:49–58. 10.1249/JES.0000000000000215 31913188

[116] Sharp MA Knapik JJ Walker LA Burrell L Frykman PN Darakjy SS. Physical fitness and body composition after a 9-month deployment to Afghanistan. Med Sci Sports Exerc. (2008) 40:1687–92. 10.1249/MSS.0b013e318176b978 18685520

[117] O'Leary TJ Gifford RM Double RL Reynolds RM Woods DR Wardle SL. Skeletal responses to an all-female unassisted Antarctic traverse. Bone. (2019) 121:267–76. 10.1016/j.bone.2019.02.002 30735797

[118] Sharma J Greeves JP Byers M Bennett AN Spears IR. Musculoskeletal injuries in British Army recruits: a prospective study of diagnosis-specific incidence and rehabilitation times. BMC Musculoskelet Disord. (2015) 16:106. 10.1186/s12891-015-0558-6 25935751 PMC4443544

[119] Milgrom C Giladi M Stein M Kashtan H Margulies JY Chisin R. Stress fractures in military recruits. A prospective study showing an unusually high incidence. J Bone Joint Surg Br. (1985) 67:732–5. 10.1302/0301-620X.67B5.4055871 4055871

[120] Wentz L Liu PY Haymes E Ilich JZ. Females have a greater incidence of stress fractures than males in both military and athletic populations: a systemic review. Mil Med. (2011) 176:420–30. 10.7205/MILMED-D-10-00322 21539165

[121] O'Leary TJ Wardle SL Rawcliffe AJ Chapman S Mole J Greeves JP. Understanding the musculoskeletal injury risk of women in combat: the effect of infantry training and sex on musculoskeletal injury incidence during British Army basic training. BMJ Mil Health. (2020) 1–5. 10.1136/jramc-2019-001347. [Epub ahead of print]. 32111683

[122] Izard RM Fraser WD Negus C Sale C Greeves JP. Increased density and periosteal expansion of the tibia in young adult men following short-term arduous training. Bone. (2016) 88:13–9. 10.1016/j.bone.2016.03.015 27046087

[123] Gaffney-Stomberg E Lutz LJ Rood JC Cable SJ Pasiakos SM Young AJ. Calcium and vitamin D supplementation maintains parathyroid hormone and improves bone density during initial military training: a randomized, double-blind, placebo controlled trial. Bone. (2014) 68:46–56. 10.1016/j.bone.2014.08.002 25118085

[124] Hughes JM Gaffney-Stomberg E Guerriere KI Taylor KM Popp KL Xu C. Changes in tibial bone microarchitecture in female recruits in response to 8 weeks of U.S. army basic combat training. Bone. (2018) 113:9–16. 10.1016/j.bone.2018.04.021 29709620

[125] Gaffney-Stomberg E Nakayama AT Guerriere KI Lutz LJ Walker LA Staab JS. Calcium and vitamin D supplementation and bone health in Marine recruits: effect of season. Bone. (2019) 123:224–33. 10.1016/j.bone.2019.03.021 30902791

[126] O'Leary TJ Izard RM Walsh NP Tang JCY Fraser WD Greeves JP. Skeletal macro- and microstructure adaptations in men undergoing arduous military training. Bone. (2019) 125:54–60. 10.1016/j.bone.2019.05.009 31077851

[127] Rauh MJ Macera CA Trone DW Shaffer RA Brodine SK. Epidemiology of stress fracture and lower-extremity overuse injury in female recruits. Med Sci Sports Exerc. (2006) 38:1571–7. 10.1249/01.mss.0000227543.51293.9d 16960517

[128] Shaffer RA Rauh MJ Brodine SK Trone DW Macera CA. Predictors of stress fracture susceptibility in young female recruits. Am J Sports Med. (2006) 34:108–15. 10.1177/0363546505278703 16170040

[129] Winfield AC Moore J Bracker M Johnson CW. Risk factors associated with stress reactions in female Marines. Mil Med. (1997) 162:698–702. 10.1093/milmed/162.10.698 9339087

[130] Knapik JJ Graham B Cobbs J Thompson D Steelman R Jones BH. A prospective investigation of injury incidence and injury risk factors among Army recruits in military police training. BMC Musculoskelet Disord. (2013) 14:32. 10.1186/1471-2474-14-32 23327563 PMC3626559

[131] Ruffing JA Nieves JW Zion M Tendy S Garrett P Lindsay R. The influence of lifestyle, menstrual function and oral contraceptive use on bone mass and size in female military cadets. Nutr Metab. (2007) 4:17. 10.1186/1743-7075-4-17 17683610 PMC1997123

[132] Lappe JM Stegman MR Recker RR. The impact of lifestyle factors on stress fractures in female army recruits. Osteoporos Int. (2001) 12:35–42. 10.1007/s001980170155 11305081

[133] Cline AD Jansen GR Melby CL. Stress fractures in female army recruits: implications of bone density, calcium intake, and exercise. J Am Coll Nutr. (1998) 17:128–35. 10.1080/07315724.1998.10718738 9550456

[134] Lauder TD Dixit S Pezzin LE Williams MV Campbell CS Davis GD. The relation between stress fractures and bone mineral density: evidence from active-duty Army women. Arch Phys Med Rehabil. (2000) 81:73–9. 10.1016/S0003-9993(00)90225-9 10638880

[135] Kelly EW Jonson SR Cohen ME Shaffer R. Stress fractures of the pelvis in female navy recruits: an analysis of possible mechanisms of injury. Mil Med. (2000) 165:142–6. 10.1093/milmed/165.2.142 10709377

[136] Armstrong DW III Rue JP Wilckens JH Frassica FJ. Stress fracture injury in young military men and women. Bone. (2004) 35:806–16. 10.1016/j.bone.2004.05.014 15336620

[137] Moran DS Heled Y Arbel Y Israeli E Finestone AS Evans RK. Dietary intake and stress fractures among elite male combat recruits. J Int Soc Sports Nutr. (2012) 9:6. 10.1186/1550-2783-9-6 22413851 PMC3382422

[138] Strohbach CA Scofield DE Nindl BC Centi AJ Yanovich R Evans RK. Female recruits sustaining stress fractures during military basic training demonstrate differential concentrations of circulating IGF-I system components: a preliminary study. Growth Horm IGF Res. (2012) 22:151–7. 10.1016/j.ghir.2012.04.007 22704365

[139] Diment BC Fortes MB Greeves JP Casey A Costa RJ Walters R. Effect of daily mixed nutritional supplementation on immune indices in soldiers undertaking an 8-week arduous training programme. Eur J Appl Physiol. (2012) 112:1411–8. 10.1007/s00421-011-2096-8 21822678

[140] Kramer TR Moore RJ Shippee RL Friedl KE Martinez-Lopez L Chan MM. Effects of food restriction in military training on T-lymphocyte responses. Int J Sports Med. (1997) 18(Suppl. 1):S84–90. 10.1055/s-2007-972704 9129267

[141] Gomez-Merino D Chennaoui M Burnat P Drogou C Guezennec CY. Immune and hormonal changes following intense military training. Mil Med. (2003) 168:1034–8. 10.1093/milmed/168.12.1034 14719632

[142] Gomez-Merino D Drogou C Chennaoui M Tiollier E Mathieu J Guezennec CY. Effects of combined stress during intense training on cellular immunity, hormones and respiratory infections. Neuroimmunomodulation. (2005) 12:164–72. 10.1159/000084849 15905625

[143] Tiollier E Chennaoui M Gomez-Merino D Drogou C Filaire E Guezennec CY. Effect of a probiotics supplementation on respiratory infections and immune and hormonal parameters during intense military training. Mil Med. (2007) 172:1006–11. 10.7205/MILMED.172.9.1006 17937368

[144] Tiollier E Gomez-Merino D Burnat P Jouanin JC Bourrilhon C Filaire E. Intense training: mucosal immunity and incidence of respiratory infections. Eur J Appl Physiol. (2005) 93:421–8. 10.1007/s00421-004-1231-1 15490219

[145] Wood SM Kennedy JS Arsenault JE Thomas DL Buck RH Shippee RL. Novel nutritional immune formula maintains host defense mechanisms. Mil Med. (2005) 170:975–85. 10.7205/MILMED.170.11.975 16450827

[146] Bernton E Hoover D Galloway R Popp K. Adaptation to chronic stress in military trainees. Adrenal androgens, testosterone, glucocorticoids, IGF-1, and immune function. Ann N Y Acad Sci. (1995) 774:217–31. 10.1111/j.1749-6632.1995.tb17383.x-i1 8597461

[147] Carins J Booth C. Salivary immunoglobulin-A as a marker of stress during strenuous physical training. Aviat Space Environ Med. (2002) 73:1203–7. 12498549

[148] Boyum A Wiik P Gustavsson E Veiby OP Reseland J Haugen AH. The effect of strenuous exercise, calorie deficiency and sleep deprivation on white blood cells, plasma immunoglobulins and cytokines. Scand J Immunol. (1996) 43:228–35. 10.1046/j.1365-3083.1996.d01-32.x 8633203

[149] Gundersen Y Opstad PK Reistad T Thrane I Vaagenes P. Seven days' around the clock exhaustive physical exertion combined with energy depletion and sleep deprivation primes circulating leukocytes. Eur J Appl Physiol. (2006) 97:151–7. 10.1007/s00421-006-0150-8 16506059

[150] Walsh NP Gleeson M Shephard RJ Gleeson M Woods JA Bishop NC. Position statement. Part one: Immune function and exercise. Exerc Immunol Rev. (2011) 17:6–63. 21446352

[151] Walsh NP Gleeson M Pyne DB Nieman DC Dhabhar FS Shephard RJ et al. Position, statement. Part two: Maintaining immune health. Exerc Immunol Rev. (2011) 17:64–103. 21446353

[152] Martinez-Lopez LE Friedl KE Moore RJ Kramer TR. A longitudinal study of infections and injuries of ranger students. Mil Med. (1993) 158:433–7. 10.1093/milmed/158.7.433 8351042

[153] Flakoll PJ Judy T Flinn K Carr C Flinn S. Postexercise protein supplementation improves health and muscle soreness during basic military training in Marine recruits. J Appl Physiol. (2004) 96:951–6. 10.1152/japplphysiol.00811.2003 14657039

[154] Whitham M Laing SJ Dorrington M Walters R Dunklin S Bland D. The influence of an arduous military training program on immune function and upper respiratory tract infection incidence. Mil Med. (2006) 171:703–9. 10.7205/MILMED.171.8.703 16933809

[155] Brenner IK Severs YD Rhind SG Shephard RJ Shek PN. Immune function and incidence of infection during basic infantry training. Mil Med. (2000) 165:878–83. 10.1093/milmed/165.11.878 11143439

[156] Li X Kan EM Lu J Cao Y Wong RK Keshavarzian A. Combat-training increases intestinal permeability, immune activation and gastrointestinal symptoms in soldiers. Aliment Pharmacol Ther. (2013) 37:799–809. 10.1111/apt.12269 23432460

[157] Riddle MS Savarino SJ Sanders JW. Gastrointestinal infections in deployed forces in the Middle East theater: an historical 60 year perspective. Am J Trop Med Hyg. (2015) 93:912–7. 10.4269/ajtmh.15-0200 26350450 PMC4703254

[158] Karl JP Hatch AM Arcidiacono SM Pearce SC Pantoja-Feliciano IG Doherty LA. Effects of psychological, environmental and physical stressors on the gut microbiota. Front Microbiol. (2018) 9:2013. 10.3389/fmicb.2018.02013 30258412 PMC6143810

[159] Karl JP Margolis LM Madslien EH Murphy NE Castellani JW Gundersen Y. Changes in intestinal microbiota composition and metabolism coincide with increased intestinal permeability in young adults under prolonged physiological stress. Am J Physiol Gastrointest Liver Physiol. (2017) 312:G559–71. 10.1152/ajpgi.00066.2017 28336545

[160] Phua LC Wilder-Smith CH Tan YM Gopalakrishnan T Wong RK Li X. Gastrointestinal symptoms and altered intestinal permeability induced by combat training are associated with distinct metabotypic changes. J Proteome Res. (2015) 14:4734–42. 10.1021/acs.jproteome.5b00603 26506213

[161] McClung JP Marchitelli LJ Friedl KE Young AJ. Prevalence of iron deficiency and iron deficiency anemia among three populations of female military personnel in the US Army. J Am Coll Nutr. (2006) 25:64–9. 10.1080/07315724.2006.10719516 16522934

[162] McClung JP Martini S Murphy NE Montain SJ Margolis LM Thrane I. Effects of a 7-day military training exercise on inflammatory biomarkers, serum hepcidin, and iron status. Nutr J. (2013) 12:141. 10.1186/1475-2891-12-141 24188143 PMC3830559

[163] McClung JP Karl JP Cable SJ Williams KW Young AJ Lieberman HR. Longitudinal decrements in iron status during military training in female soldiers. Br J Nutr. (2009) 102:605–9. 10.1017/S0007114509220873 19173765

[164] Hennigar SR Gaffney-Stomberg E Lutz LJ Cable SJ Pasiakos SM Young AJ. Consumption of a calcium and vitamin D-fortified food product does not affect iron status during initial military training: a randomised, double-blind, placebo-controlled trial. Br J Nutr. (2016) 115:637–43. 10.1017/S0007114515004766 26625709

[165] Karl JP Lieberman HR Cable SJ Williams KW Young AJ McClung JP. Randomized, double-blind, placebo-controlled trial of an iron-fortified food product in female soldiers during military training: relations between iron status, serum hepcidin, and inflammation. Am J Clin Nutr. (2010) 92:93–100. 10.3945/ajcn.2010.29185 20444958

[166] McClung JP Karl JP Cable SJ Williams KW Nindl BC Young AJ. Randomized, double-blind, placebo-controlled trial of iron supplementation in female soldiers during military training: effects on iron status, physical performance, and mood. Am J Clin Nutr. (2009) 90:124–31. 10.3945/ajcn.2009.27774 19474138

[167] Epstein D Borohovitz A Merdler I Furman M Atalli E Sorkin A. Prevalence of iron deficiency and iron deficiency anemia in strenuously training male army recruits. Acta Haematol. (2018) 139:141–7. 10.1159/000485736 29478071

[168] Booth CK Probert B Forbes-Ewan C Coad RA. Australian army recruits in training display symptoms of overtraining. Mil Med. (2006) 171:1059–64. 10.7205/MILMED.171.11.1059 17153542

[169] Yanovich R Karl JP Yanovich E Lutz LJ Williams KW Cable SJ. Effects of basic combat training on iron status in male and female soldiers: a comparative study. US Army Med Dep J. (2015) 67–73. 26101908

[170] Farina EK Taylor JC Means GE Murphy NE Pasiakos SM Lieberman HR. Effects of deployment on diet quality and nutrtional status markers of elite U.S. Army special operations forces soldiers. Nutr J. (2017) 16:41:1–9. 10.1186/s12937-017-0262-5 28673301 PMC5496422

[171] Israeli E Merkel D Constantini N Yanovich R Evans RK Shahar D. Iron deficiency and the role of nutrition among female military recruits. Med Sci Sports Exerc. (2008) 40(Suppl. 11):S685–90. 10.1249/MSS.0b013e31818946ae 18849865

[172] Moore RJ Friedl KE Tulley RT Askew EW. Maintenance of iron status in healthy men during an extended period of stress and physical activity. Am J Clin Nutr. (1993) 58:923–7. 10.1093/ajcn/58.6.923 8249880

[173] Lieberman HR Farina EK Caldwell J Williams KW Thompson LA Niro PJ. Cognitive function, stress hormones, heart rate and nutritional status during simulated captivity in military survival training. Physiol Behav. (2016) 165:86–97. 10.1016/j.physbeh.2016.06.037 27374427

[174] Pasiakos SM Margolis LM Murphy NE McClung HL Martini S Gundersen Y. Effects of exercise mode, energy, and macronutrient interventions on inflammation during military training. Physiol Rep. (2016) 4:e12820. 10.14814/phy2.12820 27273884 PMC4908496

[175] Nindl BC Leone CD Tharion WJ Johnson RF Castellani JW Patton JF. Physical performance responses during 72 h of military operational stress. Med Sci Sports Exerc. (2002) 34:1814–22. 10.1097/00005768-200211000-00019 12439088

[176] Lieberman HR Castellani JW Young AJ. Cognitive function and mood during acute cold stress after extended military training and recovery. Aviat Space Environ Med. (2009) 80:629–36. 10.3357/ASEM.2431.2009 19601505

[177] Lieberman HR Bathalon GP Falco CM Kramer FM Morgan CA III Niro P. Severe decrements in cognition function and mood induced by sleep loss, heat, dehydration, and undernutrition during simulated combat. Biol Psychiatry. (2005) 57:422–9. 10.1016/j.biopsych.2004.11.014 15705359

[178] Lieberman HR Niro P Tharion WJ Nindl BC Castellani JW Montain SJ. Cognition during sustained operations: comparison of a laboratory simulation to field studies. Aviat Space Environ Med. (2006) 77:929–35. 16964742

[179] Lieberman HR Tharion WJ Shukitt-Hale B Speckman KL Tulley R. Effects of caffeine, sleep loss, and stress on cognitive performance and mood during U.S. Navy SEAL training. Sea-Air-Land. Psychopharmacology. (2002) 164:250–61. 10.1007/s00213-002-1217-9 12424548

[180] Owen G Turley H Casey A. The role of blood glucose availability and fatigue in the development of cognitive impairment during combat training. Aviat Space Environ Med. (2004) 75:240–6. 15018292

[181] Keramidas ME Gadefors M Nilsson LO Eiken O. Physiological and psychological determinants of whole-body endurance exercise following short-term sustained operations with partial sleep deprivation. Eur J Appl Physiol. (2018) 118:1373–84. 10.1007/s00421-018-3869-0 29687266 PMC6028900

[182] Shukitt-Hale B Askew EW Lieberman HR. Effects of 30 days of undernutrition on reaction time, moods, and symptoms. Physiol Behav. (1997) 62:783–9. 10.1016/S0031-9384(97)00236-9 9284498

[183] Suurd Ralph C Vartanian O Lieberman HR Morgan CA III Cheung B. The effects of captivity survival training on mood, dissociation, PTSD symptoms, cognitive performance and stress hormones. Int J Psychophysiol. (2017) 117:37–47. 10.1016/j.ijpsycho.2017.04.002 28400246

[184] Lieberman HR Karl JP McClung JP Williams KW Cable S. Improved mood state and absence of sex differences in response to the stress of army basic combat training. Appl Psychol Health Well Being. (2016) 8:351–63. 10.1111/aphw.12075 27401942

[185] Lieberman HR Karl JP Niro PJ Williams KW Farina EK Cable SJ. Positive effects of basic training on cognitive performance and mood of adult females. Hum Factors. (2014) 56:1113–23. 10.1177/0018720813519472 25277020

[186] Lieberman HR Kellogg MD Bathalon GP. Female marine recruit training: mood, body composition, and biochemical changes. Med Sci Sports Exerc. (2008) 40(Suppl. 11):S671–6. 10.1249/MSS.0b013e31818943b3 18849867

[187] Lutz LJ Karl JP Rood JC Cable SJ Williams KW Young AJ. Vitamin D status, dietary intake, and bone turnover in female Soldiers during military training: a longitudinal study. J Int Soc Sports Nutr. (2012) 9:38. 10.1186/1550-2783-9-38 22866974 PMC3423002

[188] Lieberman HR Falco CM Slade SS. Carbohydrate administration during a day of sustained aerobic activity improves vigilance, as assessed by a novel ambulatory monitoring device, and mood. Am J Clin Nutr. (2002) 76:120–7. 10.1093/ajcn/76.1.120 12081825

[189] Tanskanen MM Westerterp KR Uusitalo AL Atalay M Hakkinen K Kinnunen HO. Effects of easy-to-use protein-rich energy bar on energy balance, physical activity and performance during 8 days of sustained physical exertion. PLoS ONE. (2012) 7:e47771. 10.1371/journal.pone.0047771 23094083 PMC3475712

[190] Cline AD Tharion WJ Tulley RT Hotson N Lieberman HR. Influence of a carbohydrate drink on nutritional status, body composition and mood during desert training. Aviat Space Environ Med. (2000) 71:37–44. 10632129

[191] Lieberman HR Bukhari AS Caldwell JA Wilson MA Mahoney CR Pasiakos SM. Two days of calorie deprivation induced by underfeeding and aerobic exercise degrades mood and lowers interstitial glucose but does not impair cognitive function in young adults. J Nutr. (2017) 147:110–6. 10.3945/jn.116.238246 27807037

[192] Karl JP Thompson LA Niro PJ Margolis LM McClung JP Cao JJ. Transient decrements in mood during energy deficit are independent of dietary protein-to-carbohydrate ratio. Physiol Behav. (2015) 139:524–31. 10.1016/j.physbeh.2014.11.068 25479571

[193] Johnson MJ Friedl KE Frykman PN Moore RJ. Loss of muscle mass is poorly reflected in grip strength performance in healthy young men. Med Sci Sports Exerc. (1994) 26:235–40. 10.1249/00005768-199402000-00015 8164542

[194] Ojanen T Kyrolainen H Igendia M Hakkinen K. Effect of prolonged military field training on neuromuscular and hormonal responses and shooting performance in warfighters. Mil Med. (2018) 183(11–12):e705–12. 10.1093/milmed/usy122 29860348

[195] Salonen M Huovinen J Kyrolainen H Piirainen JM Vaara JP. Neuromuscular performance and hormonal profile during military training and subsequent recovery period. Mil Med. (2019) 184:e113–9. 10.1093/milmed/usy176 30053107

[196] O'Leary TJ Saunders SC McGuire SJ Izard RM. Sex differences in neuromuscular fatigability in response to load carriage in the field in British Army recruits. J Sci Med Sport. (2018) 21:591–5. 10.1016/j.jsams.2017.10.018 29100827

[197] Knapik JJ Jones BH Meredith C Evans WJ Influence of a 3.5 day fast on physical performance. Eur J Appl Physiol. (1987) 56:428–32. 10.1007/BF00417770 3622486

[198] Murphy NE Carrigan CT Philip Karl J Pasiakos SM Margolis LM. Threshold of energy deficit and lower-body performance declines in military personnel: a meta-regression. Sports Med. (2018) 48:2169–78. 10.1007/s40279-018-0945-x 29949108

[199] Warr BJ Scofield DE Spiering BA Alvar BA. Influence of training frequency on fitness levels and perceived health status in deployed National Guard soldiers. J Strength Cond Res. (2013) 27:315–22. 10.1519/JSC.0b013e31827e1347 23222077

[200] Lester ME Knapik JJ Catrambone D Antczak A Sharp MA Burrell L. Effect of a 13-month deployment to Iraq on physical fitness and body composition. Mil Med. (2010) 175:417–23. 10.7205/MILMED-D-09-00192 20572474

[201] Pasiakos SM Margolis LM Orr JS. Optimized dietary strategies to protect skeletal muscle mass during periods of unavoidable energy deficit. FASEB J. (2015) 29:1136–42. 10.1096/fj.14-266890 25550460

[202] Bartlett CG Stankorb S. Physical performance and attrition among U.S. Air Force trainees participating in the basic military training fueling initiative. Mil Med. (2017) 182:e1603–9. 10.7205/MILMED-D-15-00451 28051980

[203] McGinnis KD McAdam JS Lockwood CM Young KC Roberts MD Sefton JM. Impact of protein and carbohydrate supplementation on musculoskeletal injuries in army initial entry training soldiers. Nutrients. (2018) 10:1938. 10.3390/nu10121938 30563273 PMC6315558

[204] Tharion WJ Shukitt-Hale B Lieberman HR. Caffeine effects on marksmanship during high-stress military training with 72 hour sleep deprivation. Aviat Space Environ Med. (2003) 74:309–14. 12688447

[205] Giles GE Mahoney CR Caruso C Bukhari AS Smith TJ Pasiakos SM. Two days of calorie deprivation impairs high level cognitive processes, mood, and self-reported exertion during aerobic exercise: a randomized double-blind, placebo-controlled study. Brain Cogn. (2019) 132:33–40. 10.1016/j.bandc.2019.02.003 30831453

[206] Lieberman HR Caruso CM Niro PJ Adam GE Kellogg MD Nindl BC. A double-blind, placebo-controlled test of 2 d of calorie deprivation: effects on cognition, activity, sleep, and interstitial glucose concentrations. Am J Clin Nutr. (2008) 88:667–76. 10.1093/ajcn/88.3.667 18779282

[207] Morgan CA III Hazlett G Southwick S Rasmusson A Lieberman HR. Effect of carbohydrate administration on recovery from stress-induced deficits in cognitive function: a double-blind, placebo-controlled study of soldiers exposed to survival school stress. Mil Med. (2009) 174:132–8. 10.7205/MILMED-D-58-7808 19317193

[208] Rognum TO Vartdal F Rodahl K Opstad PK Knudsen-Baas O Kindt E. Physical and mental performance of soldiers on high- and low-energy diets during prolonged heavy exercise combined with sleep deprivation. Ergonomics. (1986) 29:859–67. 10.1080/00140138608967198 3743541

[209] Lieberman HR Askew WE Hoyt RW Shukitt-Hale B Sharp MA Effects of 30 days of undernutrition on plasma neurotransmitter precursors, other amino acids, and behavior. J Nutr Biochem. (1997) 8:119–26. 10.1016/S0955-2863(97)00008-9

[210] Stahle L Stahle EL Granstrom E Isaksson S Annas P Sepp H. Effects of sleep or food deprivation during civilian survival training on cognition, blood glucose and 3-OH-butyrate. Wilderness Environ Med. (2011) 22:202–10. 10.1016/j.wem.2011.02.018 21962046

[211] Greeves JP. Physiological implications, performance assessment and risk mitigation strategies of women in combat-centric occupations. J Strength Cond Res. (2015) 29(Suppl. 11):S94–100. 10.1519/JSC.0000000000001116 26506206

